# Ultra-High Sensitivity Anisotropic Piezoelectric Sensors for Structural Health Monitoring and Robotic Perception

**DOI:** 10.1007/s40820-024-01539-6

**Published:** 2024-10-16

**Authors:** Hao Yin, Yanting Li, Zhiying Tian, Qichao Li, Chenhui Jiang, Enfu Liang, Yiping Guo

**Affiliations:** 1 State Key Laboratory of Metal Matrix Composites, School of Materials Science and Engineering, Shanghai Jiao Tong University, Shanghai, 200240 People’s Republic of China; 2 Beijing Vacuum Electronics Research Institute, Beijing, 100015 People’s Republic of China; 3 Fundamental Science On Vibration, Shock and Noise Laboratory, State Key Laboratory of Mechanical System and Vibration, School of Mechanical Engineering, Shanghai Jiao Tong University, Shanghai, 200240 People’s Republic of China

**Keywords:** Flexible piezoelectric filaments, Anisotropic, Ultra-high sensitivity, Structural health detection, Texture recognition

## Abstract

Monitoring minuscule mechanical signals, both in magnitude and direction, is imperative in many application scenarios, e.g., structural health monitoring and robotic sensing systems. However, the piezoelectric sensor struggles to satisfy the requirements for directional recognition due to the limited piezoelectric coefficient matrix, and achieving sensitivity for detecting micrometer-scale deformations is also challenging. Herein, we develop a vector sensor composed of lead zirconate titanate-electronic grade glass fiber composite filaments with oriented arrangement, capable of detecting minute anisotropic deformations. The as-prepared vector sensor can identify the deformation directions even when subjected to an unprecedented nominal strain of 0.06%, thereby enabling its utility in accurately discerning the 5 μm-height wrinkles in thin films and in monitoring human pulse waves. The ultra-high sensitivity is attributed to the formation of porous ferroelectret and the efficient load transfer efficiency of continuous lead zirconate titanate phase. Additionally, when integrated with machine learning techniques, the sensor’s capability to recognize multi-signals enables it to differentiate between 10 types of fine textures with 100% accuracy. The structural design in piezoelectric devices enables a more comprehensive perception of mechanical stimuli, offering a novel perspective for enhancing recognition accuracy.

**Supplementary Information:**

The online version contains supplementary material available at 10.1007/s40820-024-01539-6.

## Highlights

- A novel anisotropic sensor with oriented piezoelectric filaments was prepared, capable of detecting both the magnitude and direction of micro-deformations.
- Due to the efficient load transfer of continuous fibers and the formation of porous ferroelectrets, an ultra-low strain detection limit of 0.06% was achieved in the sensor.
- Given the sensor's ultra-low detection limit and deformation direction sensing capability, we developed the sensor for detecting micron-scale deformations in thin-film structures and for robotic tactile sensing applications.

**Supplementary Information:**

The online version contains supplementary material available at 10.1007/s40820-024-01539-6.

## Graphical abstract

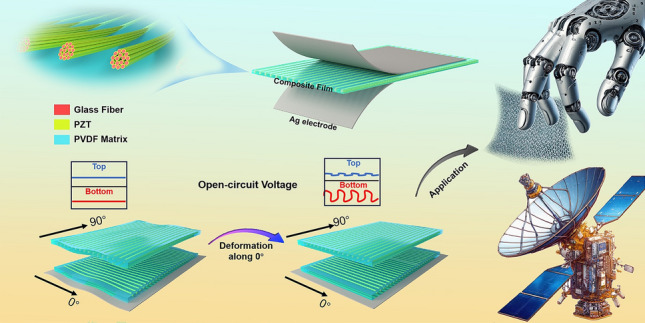

## Introduction

Sensors that can detect subtle mechanical stimuli along with their direction are highly desired for structural health detection and robotic sensory systems [1–6]. As space exploration advances, thin-film structures like antennas, solar wings, and solar sails are becoming widespread on spacecraft, leveraging their benefits of expansive area, minimal weight, and effortless deployment [7–9]. During operation, the harsh space environment can result in the abnormal deformation of the thin-film structures, leading to performance degradation and even catastrophic accidents, posing threats to life safety [10, 11]. Based on that, a flexible sensor is required to achieve the detection of anisotropic subtle structural deformation in real-time [12, 13]. On the other hand, tactile recognition systems are vital for robots to interact with their environment [14, 15]. The ability to discern textures similar to humans is a crucial aspect of these systems, enabling robots to perform intricate tasks such as inspection sampling, resource detection, and emergency rescues [16–18]. Flexible sensors capable of simultaneously identifying the roughness of textures and the direction of patterns can provide more physical information for texture recognition, thereby improving recognition accuracy.

Among the various types of sensors available, flexible piezoelectric sensors are intriguing for their capacity to convert mechanical energy into electrical signals without requiring an external power source [19, 20]. These sensors are fabricated from materials like piezoelectric ceramics [21], polymers [22], and two-dimensional materials [23] using methods such as 3D printing [24, 25], electrospinning [26], spin-coating [27] and so on. When evaluating the performance of flexible piezoelectric sensors, sensitivity, durability, response time, and multifunctionality are critical considerations. Of these, sensitivity stands out as paramount, reflecting the sensor’s ability to detect subtle mechanical stimuli [28–30]. To enhance this capability, techniques such as improving the material’s piezoelectric coefficient and enhancing mechanical transmission efficiency have been developed [31–33]. Nevertheless, achieving microscale deformation detection with piezoelectric sensors still poses a significant challenge.

On the other hand, external mechanical stimuli not only contain information about their magnitude but also their direction [1, 34–36]. The direct relationship between piezoelectric output and mechanical input relies on the piezoelectric coefficient matrix, primarily determined by the asymmetry of the crystal structure [37–39]. It is difficult to find a piezoelectric material with a suitable piezoelectric coefficient matrix to achieve vector sensing. However, deformations on thin-film structures exhibit anisotropic, and the surface textures of materials are fairly complex. These application scenarios urgently require sensors with directional perception capabilities [40–43]. Simulating the asymmetry of the piezoelectric crystal to design the macroscopic anisotropy of sensor components can effectively optimize the piezoelectric coefficients in various directions, thereby achieving sensitivity of the output signal to deformation directions [44–47]. As one of the typical anisotropic structures, fiber-shaped piezoelectric materials are characterized by their significant aspect ratios and demonstrate a notable disparity in electromechanical response between their longitudinal and radial orientations [48]. Inspired by this, we have designed an extremely sensitive vector sensor based on piezoelectric filaments.

Herein, a highly anisotropic thin film with fully oriented lead zirconate titanate-glass fiber (PZT-EGF) filaments has been engineered, capable of simultaneously identifying the magnitude and direction of micrometer-scale deformations. An immersion apparatus has been set up for the continuous preparation of PZT-EGF composite filaments using the sol–gel method, also facilitating the complete alignment of the filaments in a specified direction. After sintering, the filaments orientation was fixed using polyvinylidene fluoride (PVDF), thus achieving anisotropic piezoelectric coefficients from the longitudinal to the radial orientation of the filaments. The low detection limit of the device is mainly attributed to the presence of holes formed in the filament, which act as ferroelectret, capable of detecting strains as small as 0.06%. The continuous phase of PZT enhances load transfer efficiency, further lowering the detection limit. The corresponding sensors can be used for pulse wave monitoring, thin-film deformation inversion, and microtexture recognition, achieving high resolution capable of detecting film wrinkles as low as 5 μm in height and a surface roughness of about 20 µm.

## Experimental Section

### Preparation of the PZT [Pb(Zr_0.52_Ti_0.48_)O_3_] Sol Solution

The PZT-EGF composite filaments were fabricated by a templated-assisted sol–gel method. The 0.5 M PZT sol solution was prepared by the addition of the lead acetate trihydrate (AR, SCR), titanium butoxide (≥ 99%, Aladdin), zirconium n-propoxide (70 wt%, n-propyl alcohol solution) into 2-methoxyehanol (AR, Aladdin). In the process, an excess of lead acetate by 10% is used to compensate for the lead volatilization during high-temperature sintering. Acetylacetone (AR, Aladdin) is employed to regulate the hydrolysis rate of titanium butoxide. The particles are dissolved under magnetic stirring at 70 °C. After the solution becomes clear, the sol is allowed to age at room temperature for 48 h.

### Preparation of the PZT-Glass Fiber Composite Filaments

Composite fiber filaments are continuously prepared through a set of dip coating equipment, as shown in Fig. S3. Electronic grade glass fibers (Honghe Electronic Technology Materials Co., LTD) are drawn out from the cone, first passed through a sol solution, then dried in a heating apparatus at 160 °C, and finally wound on the Polypropylene (PP) cone. The EGF fiber was dipped coating 15 times with PZT sol by repeating the above process. Finally, the filaments were evenly arranged on an aluminum oxide ceramic sheet at a specific spacing and impregnated once more with PZT sol solution to compensate for the surface PZT gel lost due to friction during the filament drawing process. The perovskite phase of PZT is ultimately formed after sintering at 700 °C for 100 min.

### Fabrication of the Anisotropic PZT-EGF Sensor

The PVDF (Bide Pharmatech Co., Ltd) was dissolved in dimethylformamide (AR, Aladdin) to prepare a solution with a mass fraction of 20%, which is used to fix the position of the oriented PZT filaments. The ends of the PZT filaments were fixed on the ceramic sheet. Subsequently, PVDF without air bubbles was spin-coated onto the ceramic sheet at a speed of 100 rpm for 30 s, followed by curing at 70 °C for 5 h. After demolding, a flexible Ag paste was applied to a 1.5 cm × 1.5 cm area on both the both top and bottom surfaces of the composite film to serve as electrodes. The anisotropic composite sensor without voids was fabricated for comparison. The elimination of these voids within the composite filaments was achieved by evacuating the ceramic substrates to 1 MPa for 10 min before curing of the PVDF. The dual-layer vector device was constructed by orthogonally positioning two single-layer anisotropic composite films and bonding them with adhesive tape. A layer of polyimide tape was used as the outermost encapsulation layer of the device to isolate it from environmental interference. The sensor was subjected to a DC voltage poling of 40 kV cm^−1^ for 1 h at room temperature before the measurement.

### Characterization and Measurements

The morphologies and the energy-dispersive spectroscopy mapping of the PZT-EGF composite filaments were characterized by a scanning electron microscope (NERCN-TC-006). The phase structure of the PZT was examined by the X-ray diffraction (XRD, Rigaku D/MAX 2400, Cu Kα radiation). The β-phase content in PVDF was characterized by the Fourier transform infrared spectroscopy (FTIR, Nicolet 6700). The piezoresponse force microscopy (PFM) mode of the atomic force microscope (Bruker Multimode 8) was utilized for investigating the PZT-EGF fiber’s piezoresponse activity. The mechanical properties were measured using a universal testing machine (ZQ-990) with stretching velocity of 5 mm min^−1^. The output voltage was measured by a Keithley 6514 electrometer with a linear motor testing system.

## Results and Discussion

### Design of the Vector Sensor

**Fig. 1:**
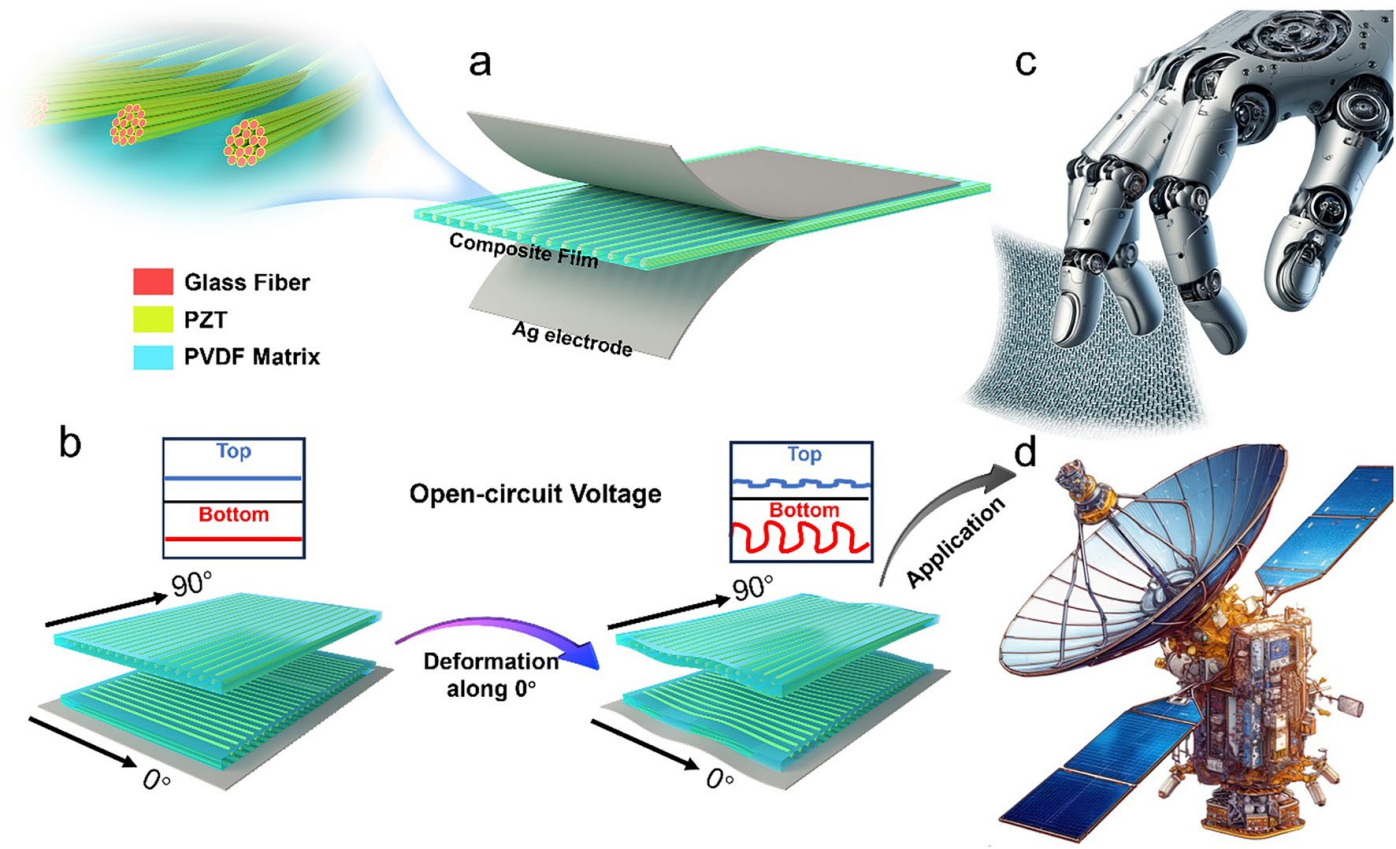
**a** Structure of the vector sensor based on PZT filaments. **b** Schematic of sensors with various filament orientations in response to 0-degree deformation. Schematic of **c** micro texture sensing system and** d** thin-film structural health monitoring

The conceptual scheme is outlined in Fig. 1. The vector sensor is constituted by a continuous array of PZT-EGF filaments aligned along the specified direction, encapsulated within a PVDF layer, and sandwiched between top and bottom Ag electrodes (Fig. 1a). These continuous EGF filament bundles are composed of multiple microfibers, with PZT growing along the surface of the microfibers in multiple layers, preserving the holes between the fibers. The formation of such porous ferroelectret, coupled with the effective load transfer efficiency of continuous PZT, endows the sensor with excellent micrometer-level deformation resolution, providing a new perspective for the study of piezoelectric sensors. As depicted in Fig. 1b, the reduction of effective piezoelectric coefficient (e^eff^) in composite film from the longitudinal direction to the radial direction of the filaments gives rise to diverse output signals along different deformation directions. Additionally, orthogonal dual-layer vector piezoelectric sensors can accurately decouple the direction and magnitude of subtle vibration stimuli. The constructed ultra-high sensitivity vector sensor holds promise in assisting tactile robots in identifying subtle surface textures and is also expected to attach to spacecraft thin-film structures for real-time monitoring of abnormal deformations (Fig. 1c, d).

### Characterization and Response Mechanism of the Vector Sensor

**Fig. 2:**
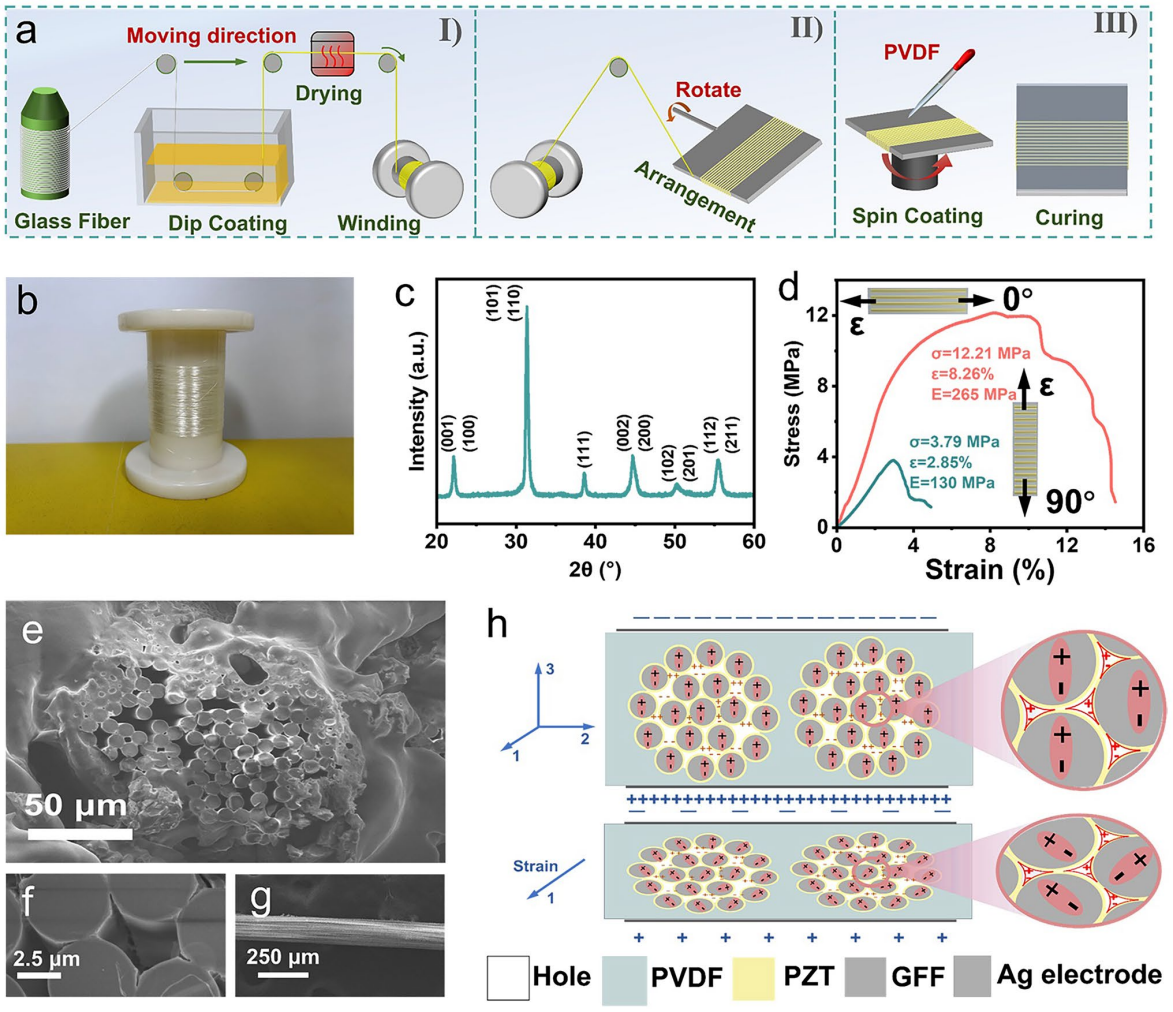
**a** Fabrication of the anisotropic piezoelectric sensor. **b** Photographs of the continuously impregnated GFF filament (over 10 m). **c** XRD pattern of the PZT-GFF composite filament. **d** Stress–strain curves of the PZT-GFF composite film along 0° and 90°. **e** SEM image of the anisotropic piezoelectric film cross-section. **f** The SEM image of the void existing in the PZT-GFF composite filament. **g** SEM image of the continuous PZT phases grown on the surface of EGF filaments. **h** Schematic diagram represents the contribution of piezoelectric output from voids and PZT

Figure 2a shows the preparation process of the vector sensor. We first set up a dip coating apparatus to grow PZT on the continuous glass fiber surface using the sol–gel method. The piezoelectric fibers were fixed in an oriented arrangement with PVDF, resulting in the anisotropic vector piezoelectric sensor (Fig. S4). The composite film exhibits excellent flexibility, with a bending radius as small as 0.94 mm (Fig. S5). Figure 2b illustrates continuous glass fiber strands impregnated with PZT precursor solution, with a total length exceeding 10 m, indicating the industrial production potential of PZT-EGF composite filaments. After sintering, the crystalline structure of the sintered PZT-EGF composite filament was examined by the XRD measurement (Fig. 2c). The perovskite-typed crystal structure without impure phase was confirmed. The homogeneously distributed Pb, Zr, Ti, and O elements as the energy-dispersive spectroscopic mapping (Fig. S6) further confirmed the crystal component of the PZT [49, 50]. The thickness of PZT on the surface of glass fibers is ~ 194 nm (Fig. S7). Additionally, PZT grows uniformly on the surface of EGF (Fig. S8). The piezoresponse and grain size of PZT grown on glass fibers are depicted in Fig. S9, characterized by PFM. Additionally, we performed piezoelectric output measurements, PFM, and FTIR tests on the PVDF matrix. The test results indicate that the piezoelectricity of the PVDF matrix curing at 70 °C is very weak, and its influence on the piezoelectric output of the device can be neglected (Figs. S10–S12).

Mechanical tensile tests were conducted on composite film strips with a width of 5 mm, featuring different filaments orientations (Fig. 2d). The experimental results substantiate that: when the film is stretched along the filament longitudinal direction (0°), the filaments and the matrix share the applied stress, resulting in the material exhibiting higher strength and modulus (12.21 MPa, 265 MPa). Conversely, when the film is stretched radially along the filaments (90°), the stress is predominantly borne by the PVDF matrix, leading to a material with lower strength and modulus (3.79 MPa, 130 MPa) [51, 52]. The electrical output is mainly produced by converting mechanical stress distributed along the piezoelectric filaments through the direct piezoelectric effect. Therefore, it is understood that the piezoelectric output varies with different strain directions. Upon reaching peak strength, we observed a segmented fracture in the composite film, ascribing to the sequential breakage of filaments.

The SEM images of the anisotropic piezoelectric thin-films cross-section reveal the existence of multiple voids, approximately 5 μm (Fig. S13) in size, between the multi-layer filaments. This suggests the formation of a porous ferroelectret structure (Fig. 2e, f). The presence of these voids is attributed to the growth of a continuous PZT phase on the surface of the glass fibers, preventing external PVDF from penetrating the fibers (Fig. 2g). When a DC voltage of 40 kV cm^−1^ electric field is applied to polarize the composite film, pairs of positive and negative charges accumulate on the inner top and bottom surfaces of the voids, forming macroscopic "quasi-dipoles". Simultaneously, the dipoles within the piezoelectric crystals align with the direction of the electric field. As indicated in Fig. 2h, when the film stretches in the filament longitudinal direction (1 direction), its positive Poisson’s ratio causes it to contract along the thickness direction (3 direction). This deformation causes both types of dipoles to shift, resulting in a greater change in the piezoelectric potential on the electrodes [53–56]. On the other hand, the continuous PZT phase is beneficial for the effective transmission of loads under bending mode, which also leads to the high sensitivity of piezoelectric sensors [57].

### Piezoelectric Response of the Single Anisotropic Piezoelectric Layer

**Fig. 3:**
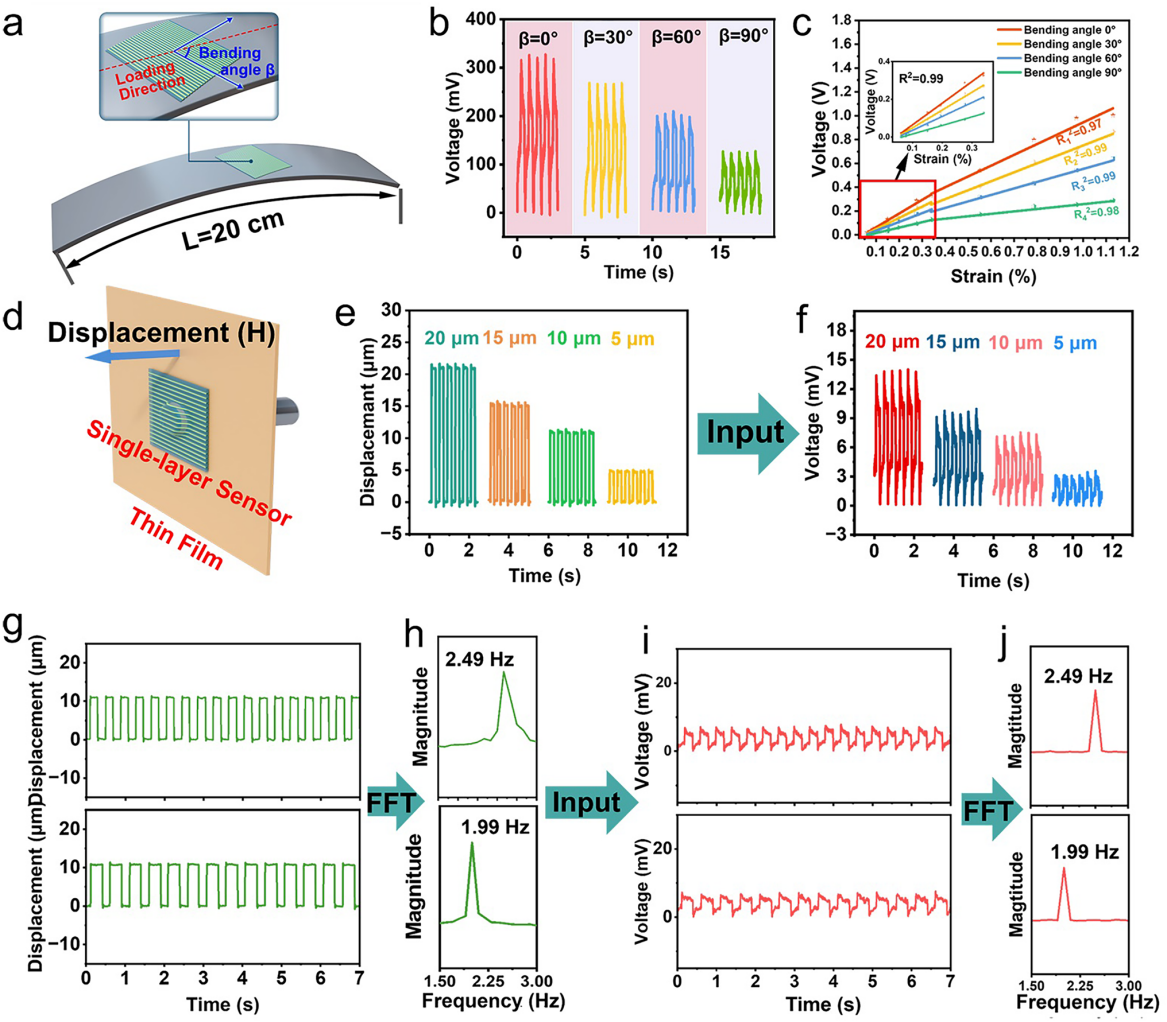
Characterization of the single-layer vector sensor.** a** Schematic diagram of micro-deformation testing for vector devices. **b** Experimental results of output voltage from the vector sensor with 0.34% strain under different bending angle. **c** Experimental results of output voltage from the vector sensor under different strain and bending angle.** d** Linear motor driving thin-film micro-protrusions schematic diagram. The thin-film micro-protrusions with **e** different magnitudes and **g** frequencies detected by the scan laser vibrometry. The corresponding real-time output voltage at different micro-protrusion **f** magnitudes and **i** frequencies. **h** Frequency spectrum generated by FFT from (g). **j** Frequency spectrum generated by FFT from (i)

We further investigated the output voltage changes in the vector sensor as a function of the micro-deformation and the bending angle (β) through experimental measurement and theoretical calculation (Fig. 3a). The device was mounted onto a PVC substrate and subjected to tensile deformation through bending, as illustrated in Fig. 3a. The mechanical parameters of the PVC substrate provided by Yiteng Plastic Sheet Co., Ltd. are shown in Table S1. The strain experienced by the sensor is equivalent to the extension of the upper surface, which is a function of the bending curvature radius [58]. To achieve subtle strains, the PVC substrate of 20 cm long is applied to create a sufficiently large curvature radius, allowing for micrometer-level strain to be generated through visibly bending the substrate. The average strain experienced by the device during bending is calculated by approximating the substrate bending shape as a smooth arc, which is ensured by the high modulus of the PVC substrate. Detailed calculations are provided in Note S1. Additionally, since the PVC substrate is 1 mm thick, significantly thicker than the ~ 150 μm piezoelectric layer, the device is positioned away from the neutral axis and remains in a stretched state during bending.

Figure 3b, c shows the experimental results of the vector sensor under different bending angles (0°, 30°, 60°, 90°) and strain (from 0.06% to 1.11%). The output voltage was recorded through a Keithley 6514 electrometer connected to a data acquisition card. At a strain level of 0.34%, the output voltage exhibits a progressive decrease from 325 to 124 mV as the bending angle increases from 0° to 90°. The output voltage is observed to follow a two-stage strong linear relationship (R^2^ > 0.97) with the applied strain, with the slope of this relationship diminishing as the strain surpasses 0.34%. This decline is attributed to the near saturation of ferroelectric polarization induced by mechanical strain at higher strain levels, which is also shown in other works [59, 60]. Consequently, the strain sensor has great advantages in detecting tiny strains. Moreover, the slope of the voltage variation with strain decreases as the bending angle increases. This trend can be ascribed to the gradual decrease in effective piezoelectric coefficients along the direction of increasing bending angles. The following correlation between the effective piezoelectric coefficient and the bending angle was established through the integration of individual filaments in the thin film, which aligns with the experimental results. The ideal open-circuit voltage ${V}_{oc}$ can be calculated as [61, 62]:

1 $$\begin{array}{*{20}c} {V_{oc} = \frac{Q}{C} = \frac{{AD_{n}^{eff} }}{C} = \frac{{A\varepsilon e_{3bq}^{eff} }}{C} } \\ \end{array}$$

where *Q* denotes the charge collected at the piezoelectric device, C represents the capacitance of the piezoelectric device, *A* is the effective device area, ${D}_{n}^{eff}$ is the electric displacement, ${e}_{3bq}^{eff}$ represents the effective piezoelectric coefficient along different angles, and $\varepsilon$ is the uniaxial strain of the composite film. Given the negligible piezoelectricity of the PVDF matrix, as shown by piezoresponse force microscopy (Figs. S10-S12), we attribute the entire effective piezoelectric coefficient to the PZT-GFF filaments. The effective piezoelectric coefficient along the bending angle direction can be expressed as:

2 $$\begin{array}{*{20}c} {e_{3bq}^{eff} = \frac{nAL}{V}e_{31} \cos \beta } \\ \end{array}$$

where *n*, *A* and *L* refer to the number, effective cross-sectional area, and length of the piezoelectric filaments, ${\text{e}}_{31}$ donates the piezoelectric coefficient of the PZT, and the *V* is the volume of the composite film. $\beta$ represents the bending angle of the device. It should be noted that the piezoelectric coefficient of PVDF is negligible during calculation, and the detailed calculations are presented in Note S2. Combining Eq. (1) and Eq. (2), and expressing all constants as *B*, the correlation between the output voltage, strain, and bending angle is delineated as follows:

3 $$\begin{array}{*{20}c} {B = \frac{{nA^{2} Le_{31} }}{CV}} \\ \end{array}$$

4 $$\begin{array}{*{20}c} {V_{oc} = B\varepsilon \cos \beta } \\ \end{array}$$

This model accounts for the observed reduction in piezoelectric sensitivity as bending angle varies, providing a quantitative understanding of the vector sensing characteristics under mechanical deformation. The pronounced deformation detection limit (strain = 0.06%) highlights the vector sensor’s proficiency in detecting ultra-microscopic vibrations, which can be attributed to the structure of the multiple holes and continuous PZT phase. Variations in arterial blood pressure throughout a heartbeat can be delicately detected using the vector sensor, confirming the sensor’s extremely low detection threshold (Fig. S14).

With the aid of the vector sensor, the subtle vibration stimulus can be captured. Consequently, such sensors can be adhesive to thin films for the inversion of microscale deformation, serving as a tool for detecting mechanical damage in the thin-film mechanical structures. This capability enables timely diagnosis of issues, thereby preventing catastrophic failures.

We developed a setup to induce microscale vibrations and deformations in thin films (Fig. 3d). Using a precision linear actuator, we generated micrometer-scale reciprocating motion along a single axis. The plastic model is attached to the front end of the actuator, making direct contact with the center of a 0.8 mm thick silicon-based thin film. The film is fixed on an acrylic plate with a hole in the middle, where the shape of the hole matches the shape of the plastic model. When the actuator operates, it causes the film to produce a reciprocating protrusion. By manipulating the geometry of the model and the hole, specific shapes of wrinkles can be generated. For instance, a 6 mm diameter cylindrical plastic tip was employed to produce circular protrusions on the film. The flexible sensor was adhered to the film to detect protrusive deformations within the size range of the device, as shown in Fig. 3d.

The reliability of the linear actuator’s displacement was meticulously validated using laser vibrometry, as depicted in Fig. 3e, g. The linear actuator exhibited the capacity to induce deformations ranging from 5 to 20 μm across various frequencies in the thin film. Figure 3f illustrates the sensor output voltage waveform corresponding to the deformations in the thin film within the 5–20 µm range. It is evident that the sensor’s capability to discern surface morphological changes exceeds an accuracy of 5 μm, with a detection threshold set at 5 μm (Video S1). However, the detection threshold of piezoelectric devices without holes is of 100 μm (Fig. S15), confirming that holes significantly increase the output performance of the device. The detailed movement trajectory of the thin film over several complete cycles at varying frequencies is presented in Fig. 3g. The observed output voltages reflected periodic fluctuations that were in alignment with the multiplicity of frequencies (Fig. 3i), underscoring the vector sensor’s capability for synchronized and reproducible detection. To further analyze these waveforms, a fast fourier transform (FFT) was applied (Fig. 3h, j), which is a computationally optimized technique for transitioning signals from the temporal to the frequency domain, essential for dissecting signals with complex frequency content. The FFT-derived frequency spectra demonstrated significant consistency and precision between the input and the output waveforms, validating the vector sensor’s ability to accurately identify subtle vibrations at various frequency bands. Additionally, a series of characterizations of the sensor’s fundamental performance were conducted, including dynamic response, response time, impact sensitivity, and durability (Fig. S16). When compared with other piezoelectric sensors and other type sensors reported in the literature, this vector sensor demonstrates superior performance, especially in sensitivity (Tables S2 and S3).

### Decoupling Bending Angle and Strain with a Double-Layer Vector Sensor

The anisotropic nature of the piezoelectric layer imparts a directional sensitivity to the sensor. However, the output voltage is influenced by a combination of the bending angle and the extent of the strain. To simultaneously discern the magnitude and the direction of bending, we utilize a biaxial sensor configuration, consisting of two orthogonally arranged vector sensors [45]. This arrangement enables the detection of micron order vibrations with precision, as each layer responds to the directional bending, providing a comprehensive assessment of the mechanical deformation.

**Fig. 4:**
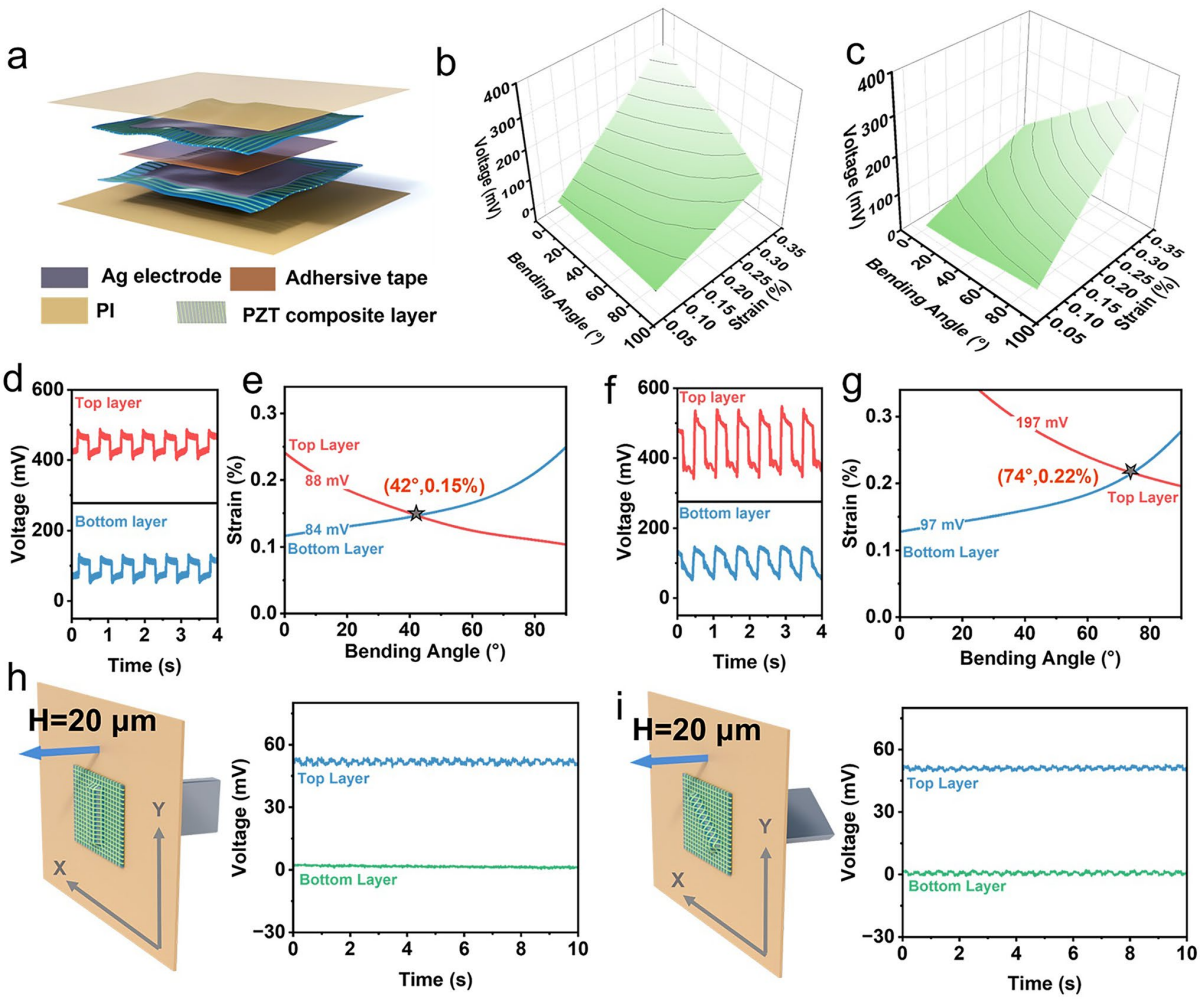
Characterization of the double-layer vector sensor.** a** Schematic of the double-layer vector sensor. The fitting results of the output voltage from **b** the bottom layer sensor and **c** the top layer sensor based on the experimental results. **d** Output voltage of the double-layer vector sensor with strain of 0.17% under 45° bending. **e** Bending angle and the strain decoupled from the output voltage in (d). **f** Output voltage of the double-layer vector sensor with strain of 0.21% under 70° bending. **g** Bending angle and the strain decoupled from the output voltage in (f). The schematic diagram of **h** 0-degree long strip, **i** 45-degree long strip wrinkle and the corresponding output response from the double-layer vector sensor

Figure 4a shows the structure of the double-layer vector sensor, consisting of the orthogonally arranged anisotropic films, an adhesive layer, and a polyimide (PI) encapsulation layer, with both layers of the sensor having the same polarization orientations. The mechanical performance of the adhesive layer is shown in the Fig. S17. We provided a theoretical understanding of the sensor’s behavior under various bending conditions, as follows:

5 $$\begin{array}{*{20}c} {V_{top} = B_{top} \varepsilon \cos \beta } \\ \end{array}$$

6 $$\begin{array}{*{20}c} {V_{bottom} = B_{bottom} \varepsilon \cos \left( {90 - \beta } \right) } \\ \end{array}$$

where ${V}_{top}$ and ${V}_{bottom}$ represent the output voltage of the top layer and bottom layer, respectively. ${B}_{top}$ and ${B}_{bottom}$ are the constant. The corresponding bending angle and strain are deduced as shown in Eqs. (7) and (8):

7 $$\begin{array}{*{20}c} {\beta = \arctan \left( {\frac{{B_{top} V_{bottom} }}{{B_{bottom} V_{top} }}} \right) } \\ \end{array}$$

8 $$\begin{array}{*{20}c} {\varepsilon = \frac{{V_{top} }}{{B_{top} \cos \beta }} } \\ \end{array}$$

It is evident that the output voltages from the upper and lower sensors display opposing trends across the 0°-90° bending range. Given that the system of equations represented by Eqs. (5) and (6) has a unique solution within the 0°–90° range, the bending radius and strain can be concurrently determined from the output voltages of double-layer sensors as subjected to bending. According to Eqs. (5) and (6), the characteristics of the double-layer vector sensor based on the experimental data were fitted in Fig. 4b, c. The output voltage is measured by testing the device while it is bent on a PVC substrate, as shown in Fig. 3a. We further verify the universality of the analyzing method. The device was adhered to another substrate with a thickness of 0.8 mm and a length of 15 cm. Using a linear motor to bend the substrate, two sets of deformations were produced on the sensor: one with a bending angle and strain of 45° and 0.18%, and another with 70° and 0.23% (Fig. 4d, f). By obtaining the output voltages from the top and bottom layers of the device and employing an image-based method to solve Eqs. (5) and (6), the calculated bending angles and strains were 42° and 0.15%, and 74° and 0.22%, respectively, which are consistent with the actual values (Fig. 4e, g).

With the aid of the double-layer vector sensor, the anisotropic deformation direction and magnitude of thin film can be captured simultaneously. Using the device shown in Fig. 3d to induce deformation in the film, the shape of the plastic model at the front end is changed to a rectangular shape to achieve anisotropic deformation in the film. When the 20 μm elongated protrusions are oriented at a 90-degree angle, they induce a stretch in the device along the same direction, resulting in no voltage output from the lower sensor layer, while the upper layer exhibits a voltage output of ~5 mV (Fig. 4h and Video S2). Conversely, when the 20 μm protrusions are oriented at a 45-degree angle, they induce a stretch in the device along the 45-degree direction, leading to nearly identical signal outputs from both the upper and lower sensor layers, each with a voltage of ~2 mV (Fig. 4i).

### Texture Recognition of the Single-Layer Vector Sensor

**Fig. 5:**
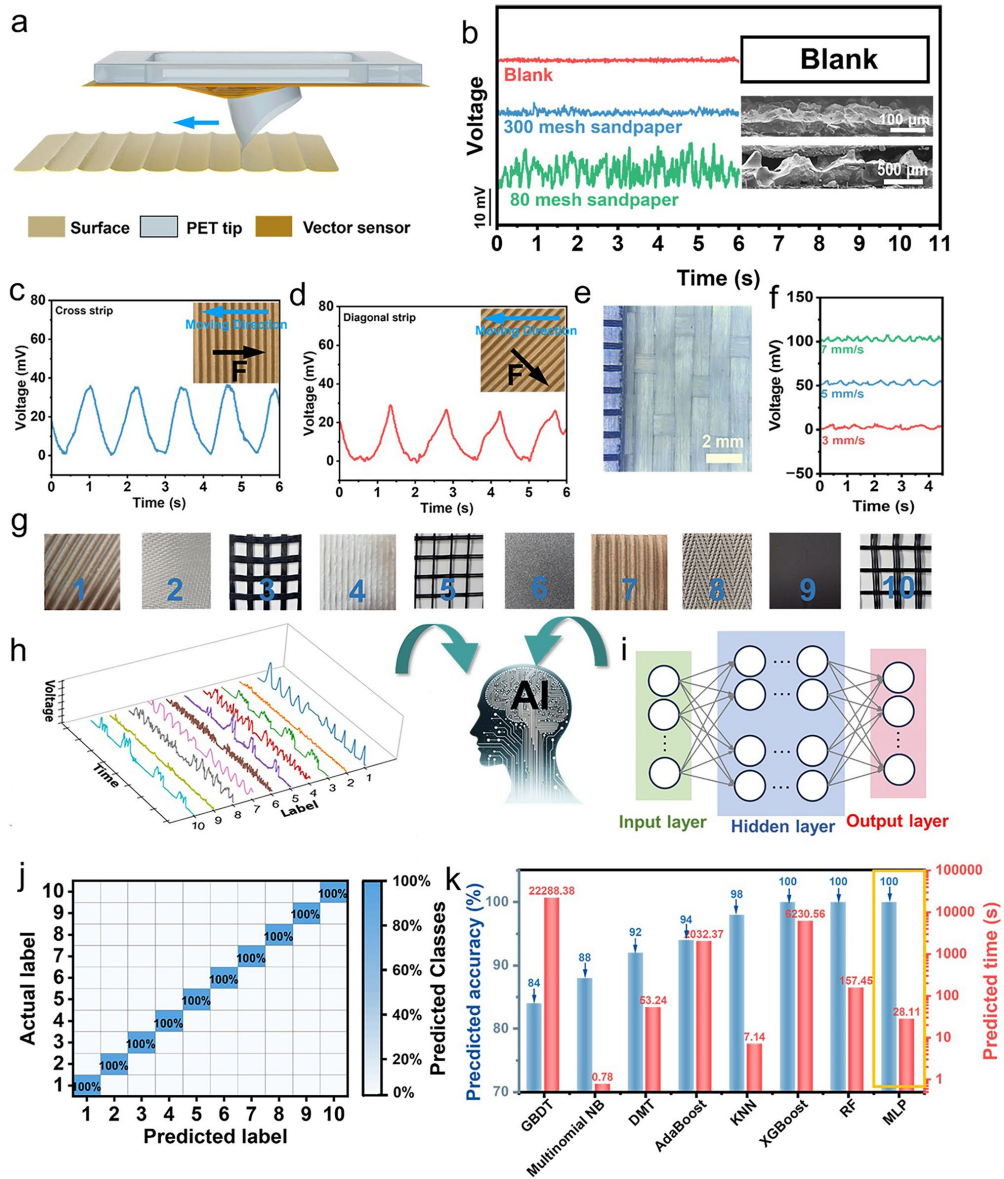
**a** Schematic of the sensory system based on the vector sensor. **b** Signals generated by the vector sensor on the textures with different roughness. The output signals by sliding the vector sensor on the **c** cross and **d** diagonal stripes. **e** Fabric image through optical microscope magnification (× 20). The insert figures in (c) and (d) illustrates the sensing mechanism for both cross and diagonal stripes, respectively. **f** Output signals by sliding the sensor on the fabric with different sliding rate (3, 5, 7 mm s^−1^). **g** Optical images of textures for perception and corresponding numbers. **h** Output signals for the textures. **i** Schematic of neural network model. **j** Confusion matrix for the recognition of the 10 different textures through the sensory system. **k** Comparison of the accuracy and computation time among different algorithms

Tactile perception is an urgent necessity in the field of robotics for enabling effective interactions with the environment. The vector sensor investigated in this study, with its high sensitivity and directional sensing capabilities, can identify multiple dimensions of texture information, including roughness and orientation. Consequently, we have successfully employed machine learning in conjunction with the vector sensor to distinguish ten distinct textures, including sandpapers with micrometer-level roughness, fibrous fabrics, and textures with varying orientations. The recognition mechanism is shown in Fig. 5a. The vector sensor was fixed on the movable frame and the PET tip was engineered to replicate the human fingerprint to capture the vibrational stimuli during contact. When this tip is moved across the surface at a set speed, it experiences varying pressures due to the different heights of the surface textures, thereby inducing vibrations on the vector device. Textures with different orientations and roughness will apply strains of varying directions and magnitudes to the sensor through the tip, resulting in different output voltages. We have designed the tip with a thickness of 100 μm and a height of 5 mm. Note that the height between the sensor and the fabric is maintained consistently in case those waveforms from different textures cannot be compared with discrepancies. Even the most minute surface features, including textures as small as tens of micrometers in height, can be detected by the vector sensor due to its high sensitivity.

We first tested the device’s output response when sliding over sandpapers of varying roughness at a speed of 7 mm s^−1^. The texture height of the 80-grit sandpaper is approximately 100 µm, while that of the 300-grit sandpaper is around 20 µm. Compared to the blank control at the top of Fig. 5b, both exhibit significant disordered piezoelectric responses corresponding to uneven textures. In addition, the larger deformation on the vector sensor with the texture height increased, leading to greater piezoelectric voltage of the 300-grit sandpaper. The directional sensitivity of this vector sensor can be utilized to differentiate surfaces with identical texture heights but varying orientations. In Fig. 5d, the cardboard is positioned at a 45-degree tilt from the state shown in Fig. 5c. The force exerted by the texture on the tip in Fig. 5c is along the 0-degree direction, while in Fig. 5d, it is along the 45-degree direction, causing the device to deform along the 45-degree axis. As a result, the output voltages vary even at the same texture height. The regular voltage waveforms of both cardboards during a 7 mm s^−1^ slide are depicted in Fig. 5c, d, and the corresponding FFT frequency spectra are applied for analysis (Fig. S18). It can be observed that the waveform for the 45-degree oriented texture has a smaller amplitude and frequency, which is due to an increase in the texture period by a factor of the $\sqrt{2}$.

We examined the sensor’s response to varying frequencies by sliding it across the fabric surface shown in Fig. 5e at speeds of 3, 5, and 7 mm s^−1^. The fabric’s texture period is 3 mm. Theoretically, the output signal frequency is jointly determined by the sliding speed and the texture period of the fabric, as follows:

9 $$\begin{array}{*{20}c} {{\text{f = }}\frac{{\text{v}}}{{\text{l}}} } \\ \end{array}$$

where *f* represents the frequency, *v* is the sliding rate and the *l* is the texture period. In practice, the output voltage waveforms at these different speeds revealed characteristic frequencies that correspond to the expected values (Fig. 5f). Therefore, various surface morphologies can be differentiated by the vector sensor due to both its high sensitivity and strain direction recognition.

The texture perception system has been integrated with machine learning algorithms to classify ten different textures (Fig. 5g). The sensor conducted 25 identical tests on each material, ensuring consistent sampling points and test durations. The compilation of these responses formed a dataset, where each entry comprised 16,833 data features. Due to the significant variability in sensor responses across materials, data preprocessing steps were conducted. Initially, the data was normalized to the range [0, 1] to standardize feature scales. Subsequently, we applied L2 norm normalization to the data, which mitigated feature disparities and bolstered the model’s stability and ability to generalize to unseen data. This preprocessing stage streamlined the model training process and enhanced its robustness. The preprocessed waveforms of the recognition signals for a single cycle of ten fabrics are depicted in Fig. 5h.

The preprocessed dataset was utilized for machine learning algorithm processing. The dataset was randomly divided into a training set (80%) and a test set (20%). For neural network models, the dataset labels were encoded using one-hot encoding. Across all machine learning models, we employed hyperparameter optimization strategies. Within our defined parameter space, we leveraged the “RandomizedSearchCV” method from the sklearn library, along with fivefold cross-validation, to optimize algorithm parameters, ensuring a balance between calculation efficiency and accuracy. We employed eight algorithms for signal classification, among which the Multilayer Perceptron (MLP) algorithm, featuring a neural network with an input layer, three fully connected hidden layers (with neuron counts of 20, 40, and 20, respectively), and an output layer, demonstrated optimal performance (Fig. 5i). It produced a confusion matrix within a short time frame of 15.98 s, ensuring 100% recognition accuracy (Fig. 5j). Figure 5k compares the accuracies and prediction times of the other algorithms. This high recognition accuracy can be attributed to two main factors. One is for the high sensitivity of the vector sensor, which allows it to discern differences in texture height and orientation with high reproducibility. The second point can be attributed to the appropriate model construction during the machine learning computation.

## Conclusion

The architectural innovation in piezoelectric sensors facilitates their acute sensitivity to both subtle deformations and their directional orientation. Achieved through a facile production method, continuous PZT-EGF composite filaments with inherent pores are oriented to form anisotropic piezoelectric films. As a result of the formation of porous ferroelectret materials and a continuous-phase PZT, the resultant composite thin film distinguishes itself with outstanding sensitivity. Furthermore, the oriented arrangement of the piezoelectric filaments endows the film with anisotropic piezoelectric coefficients. It can detect micrometer-scale deformations of the film’s protrusions and can be integrated into texture perception systems, demonstrating excellent discernment capabilities for the direction and roughness of minute textures. Moreover, when combined with machine learning neural network algorithms, it achieves a 100% resolution rate for distinguishing among 10 different surface textures. Sensors of such elevated sensitivity, minimal detection thresholds, and adeptness in directional stress discernment are poised to revolutionize structural health monitoring and intelligent robotics, heralding new technology for industrial advancement.

## Supplementary Information

Below is the link to the electronic supplementary material.

Supplementary file1 (DOCX 9683 KB)
