# Supplementary material for: Ultra-High Sensitivity Anisotropic Piezoelectric Sensors for Structural Health Monitoring and Robotic Perception
Source: Nanomicro Lett. 2024 Oct 16;17:42. doi: 10.1007/s40820-024-01539-6 (PMC11485280; doi:10.1007/s40820-024-01539-6)
Supplement: Supplementary file 1 — Supplementary file1 (DOCX 9683 KB) [file 40820_2024_1539_MOESM1_ESM.docx]

**Supporting Information**

**Ultra-High Sensitivity Anisotropic Piezoelectric Sensors for Structural Health Monitoring and Robotic Perception**

*Hao Yin^1^, Yanting Li^1^, Zhiying Tian^2^, Qichao Li^1^*, Chenhui Jiang^1^, Enfu Liang^3^, and Yiping Guo^1^**

* Qichao Li, [liqichao@sjtu.edu.cn](mailto:liqichao@sjtu.edu.cn); Yiping Guo, ypguo@sjtu.edu.cn

^1^State Key Laboratory of Metal Matrix Composites, School of Materials Science and Engineering, Shanghai Jiao Tong University, Shanghai 200240, People’s Repulic of China

^2^Beijing Vacuum Electronics Research Institute, Beijing 100015, People’s Repulic of China

^3^Fundamental Science on Vibration, Shock and Noise Laboratory, State Key Laboratory of Mechanical System and Vibration, School of Mechanical Engineering, Shanghai Jiao Tong University, Shanghai 200240, People’s Repulic of China

**Note S1 The Lambert-Beer law for β phase content calculation**

$$F_{\beta}=\frac{A_{\beta}}{\left( \frac{K_{\beta}}{K_{\alpha}} \right)A_{\alpha}+A_{\beta}} (S1.1)$$

$F_{\beta}$ is the β phase content of in the PVDF. *A*_α_ and *A*_β_ are absorbances of 762 cm^-1^ and 840 cm^-1^_._ *K*_α_ and *K*_β_ are absorption coefficients of 762 cm^-1^ and 840 cm^-1^_,_ *K*_α_=6.1×10^4^ cm^2^ mol^-1^, *K*_β_=7.7×10^4^ cm^2^ mol^-1^.

**Note S2 The method to determine the strain of PZT PENG.**

Since the thickness and modulus of PVC substrate is much larger than that of PZT composite film, it can be considered that a uniform tensile strain (ε) is applied in PZT films. Hence, ε can be expressed as:

$$\varepsilon=\frac{t}{2R} (S2.1)$$

where t is the thickness of the PVC substrate. R is the bending radius and can be obtained from the following formula as:

$$Rsin\frac{\theta}{2}=\frac{L-\triangle L}{2} (S2.2)$$

$$R\theta=L (S2.3)$$

The relevant parameters are identified in the Figure S1 (Supporting Information).

**Note S3 Theoretical prediction of the efficient piezoelectric constants**

Below we derive expression of effective piezoelectric charge constant *e* as a function of different directions from 0° to 90°. We started from analysis of a single piezoelectric filament and established a local coordinate system (k-l coordinate system) and the global coordinate system (b-q coordinate system). The coordinate axis k is parallel to the alignment direction of the fibers, while the coordinate axis b aligns with the direction of the effective piezoelectric coefficient to be calculated. The angle between the two coordinate systems is β (Figure S2, Supporting Information). The effective electric displacement and effective stress are:

$$D_{n}^{eff}=\frac{1}{V}\sum_{i=1}^{N} \int_{Vi} D_{n}^{(i)}dV_{i} (S3.1)$$

$$\varepsilon_{BQ}^{eff}=\frac{1}{V}\sum_{i=1}^{N} \int_{Vi} \sigma_{Bb}\sigma_{Qq}\varepsilon_{bq}^{(i)}dV_{i} (S3.2)$$

where $\varepsilon_{bq}^{(i)}$ and $D_{n}^{(i)}$ (b, q= 1,2) are, respectively, the strain matrix and electric displacement vector of each filament in the global coordinate system, 𝑉 and 𝑉𝑖 are, respectively, the volume of the device and of the piezoelectric filaments. According to piezoelectric equation, $e_{3bq}^{eff}$ in matrix becomes

$$e_{3bq}^{eff}=\frac{D_{3}^{eff}}{\varepsilon_{bq}} (S3.3)$$

Where $e_{3bq}^{eff}$ represents the piezoelectric coefficient along b axis. Given that the piezoelectric coefficient of PVDF in the composite film is negligible due to its small magnitude (Fig. S10-S11, ESI), the overall piezoelectric coefficient of the film can be approximated as the sum of the contributions from individual filament. To elucidate the variation of the piezoelectric coefficient within a single filament, we consider the angle β between the direction of the effective piezoelectric coefficient to be calculated and the orientation of the filaments. The two orthogonal coordinate systems are established: the k-l local coordinate system and the b-q global coordinate system. We assume that the strain $\varepsilon_{kl}^{i}$ is applied along the direction of the b-axis. The correlation between the strain matrix in the local coordinate system and the global coordinate system is achieved through the transformation matrix 𝑁𝑖 as:

$$\varepsilon_{bq}^{i}=N_{i}\varepsilon_{kl}^{i}N_{i}^{T} (S3.4)$$

Similarly, the electrical displacement contribution of each filament can be expressed as:

$$D_{3}^{(i)}=e_{3kl}\varepsilon_{kl}^{(i)}=e_{3bq}\varepsilon_{bq}^{(i)}=e_{3bq}N_{i}\varepsilon_{kl}^{i}N_{i}^{T} (S3.5)$$

where $e_{3kl}$, $\varepsilon_{kl}^{(i)}$, $e_{3bq}$ and $\varepsilon_{bq}^{(i)}$ are piezoelectric coefficient matrix and strain matrix in local coordinate system and global coordinate system, respectively. Hence, we derived the expression of the transformation matrix 𝑁𝑖 correlating the local coordinate system to the global coordinate system to calculate the effective electrical displacement $D_{n}^{eff}$and effective strain $e_{BQ}^{eff}$, and further, calculate the effective charge constants.

According to Euler's rotation theorem, any coordinate system transformation may be described using a single angle. The transformation matrix from k-l to b-q coordinate system to can be written as:

$$Ni=\left( \begin{matrix} \cos\beta& -\sin\beta\\ \sin\beta& \cos\beta\end{matrix} \right)(S3.6)$$

$$\varepsilon_{kl}^{i}=\left( \begin{matrix} \varepsilon\cos\beta& \varepsilon sin \beta\\ \varepsilon\sin\beta& 0 \end{matrix} \right) (S3.7)$$

$$\boldsymbol{D}_{\boldsymbol{3}}^{\mathbf{(}\boldsymbol{i}\mathbf{)}}\mathbf{=}\frac{\boldsymbol{AL}}{\boldsymbol{V}_{\boldsymbol{i}}}\begin{matrix} {\mathbf{(}\boldsymbol{e}}_{\mathbf{31}} & \boldsymbol{e}_{\mathbf{32}}\mathbf{)} \end{matrix}\left( \begin{aligned} \boldsymbol{\varepsilon}_{\boldsymbol{11}} \\ \boldsymbol{\varepsilon}_{\boldsymbol{22}} \end{aligned} \right)\mathbf{(}\boldsymbol{S}\mathbf{3.8)}$$

Where $n$, L, A represent the number, length and sectional area of the piezoelectric filaments. Additionally, e_31_ is equal to e_32_ in PZT phase. By substituting the Equation (S3.6) and (S3.7) into the Equation (S3.5), one can establish a functional relationship between the piezoelectric coefficients and the direction and magnitude of the strain. As follows,

$$e_{3bq}^{eff}=\frac{nAL}{V}e_{31}\cos\beta(S3.9)$$

$$B=\frac{nA^{2}Le_{31}}{CV} (S3.10)$$

$$V_{oc}=\frac{Q}{C}=\frac{AD_{n}^{eff}}{C}=\frac{A\varepsilon e_{3bq}^{eff}}{C}=B\varepsilon\cos\beta(S3.11)$$

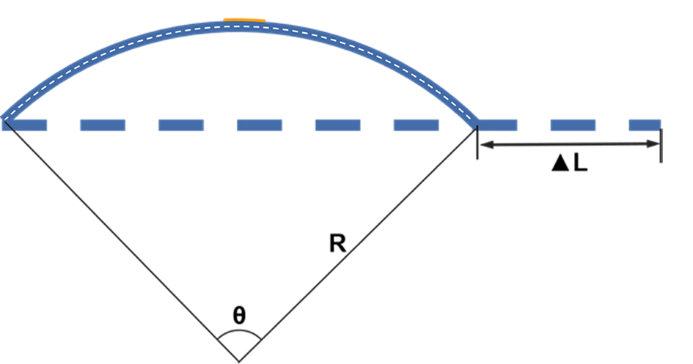


**Fig. S1** The bending test of the PZT composite film.


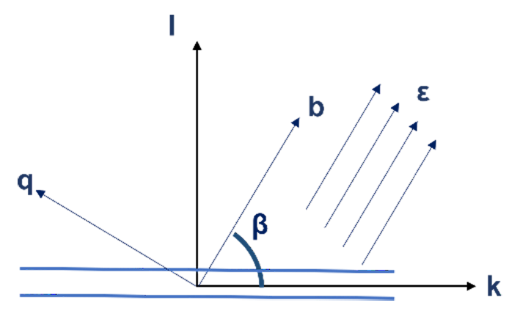


**Fig. S2** The unit of the PZT composite film.

**
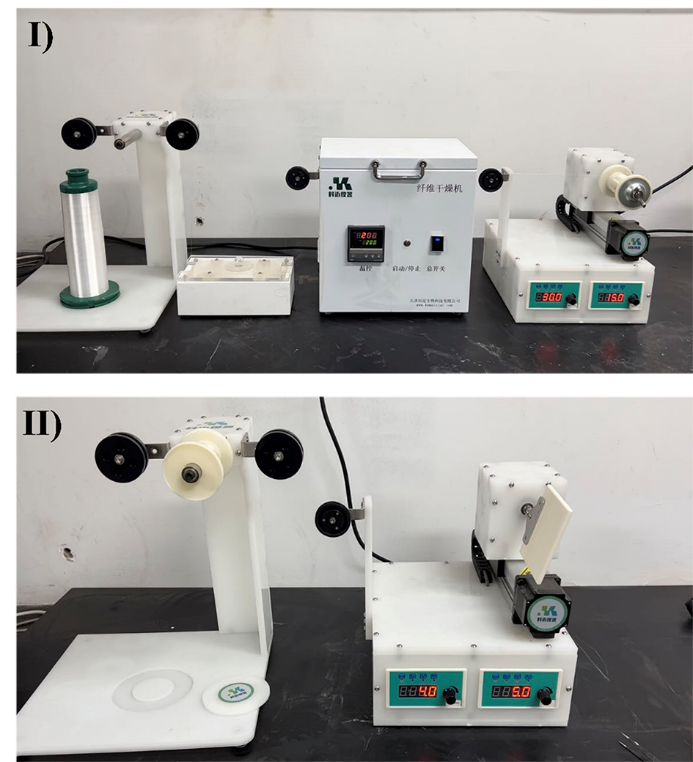
**

**Fig. S3** The optical images of the fabrication process. Ⅰ) The process of continuously dip coating. Ⅱ) The process of the filaments arrangement.

**
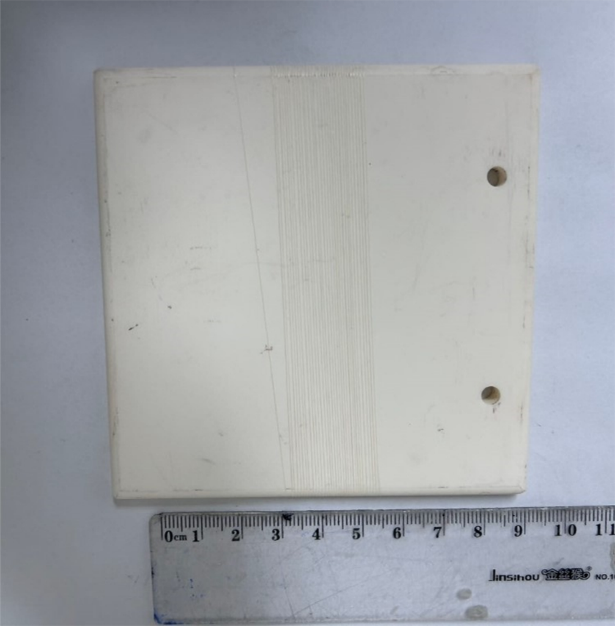
**

**Fig. S4** The oriented filaments arranged on the ceramics sheet.


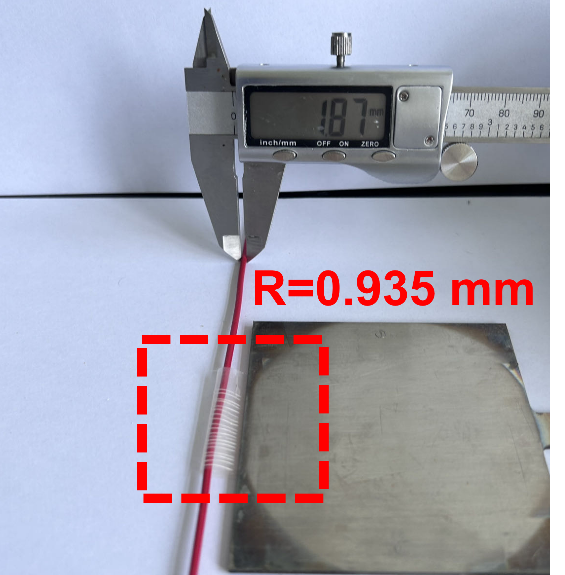


**Fig. S5** The flexibility of the piezoelectric composite film.


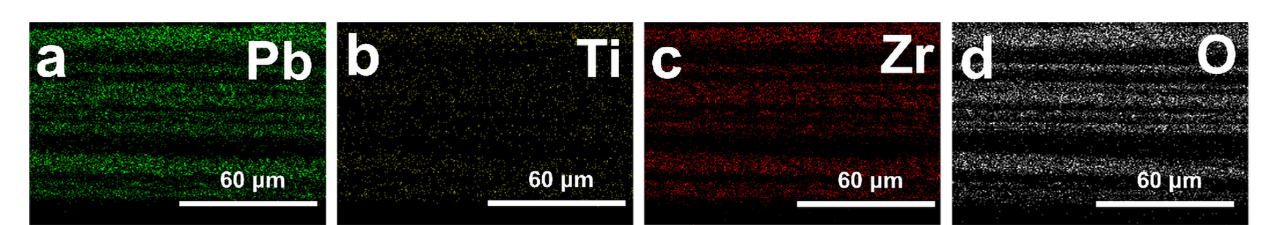


**Fig. S6** The energy-dispersive spectroscopic mapping of the a) Pb, b) Ti, c) Zr, d) O element. The distribution of different elements was obtained by EDS mapping on the surface of the PZT-EGF composite filaments, indicating that the distribution of Zr, Ti, Pb, and O elements is uniform.


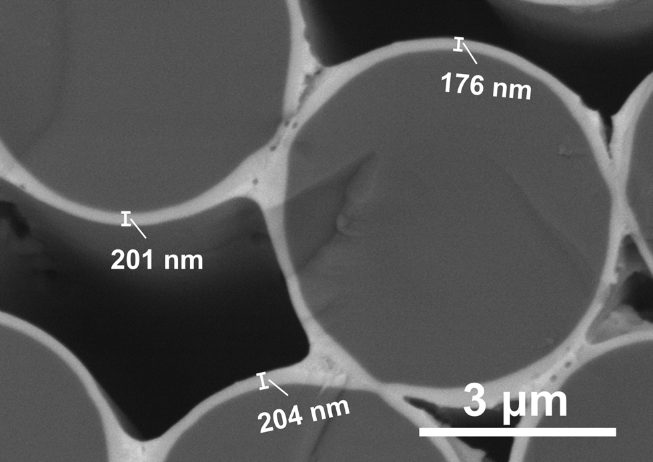


**Fig. S7** The cross section SEM image of the PZT-EGF composite filament, revealing the multistrands structure of the filament and an average PZT deposition thickness of 194 nm.

**
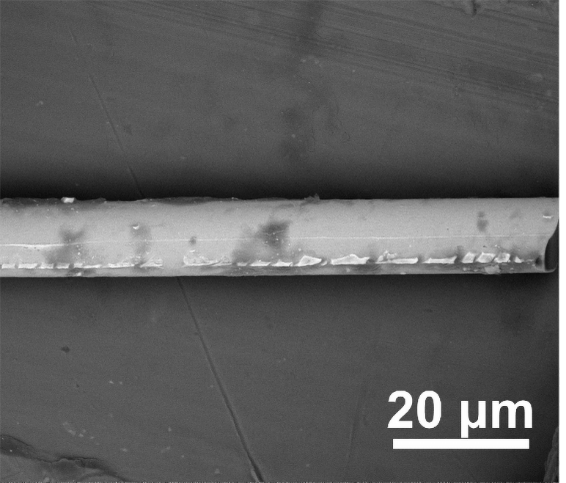
**

**Fig. S8** SEM image of the individual fiber within the PZT-EGF fiber bunbles


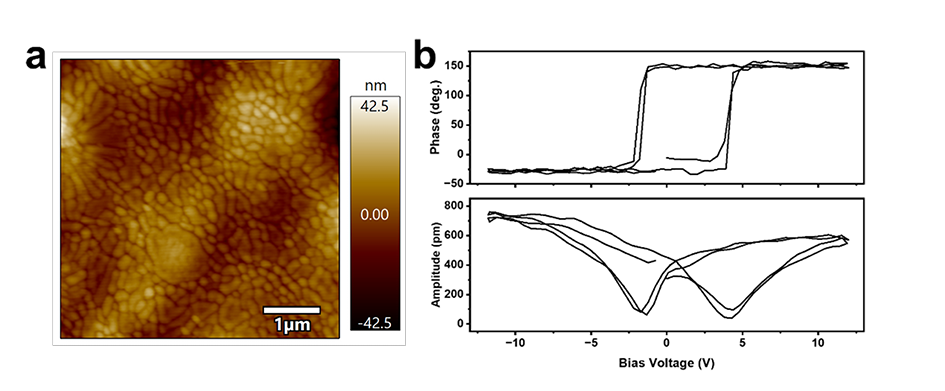


**Fig. S9** The Characterization of PZT-EGF composite filaments by atomic force microscopy (AFM). a) AFM topography image of the PZT-EGF composite filaments in 5 *5μm area, which clearly reveals the internal polycrystalline structure. b) PFM phase (top panel) and amplitude (bottom panel) of the PZT-EGF composite filaments. When subjected to 12 V, PZT composite filaments display a 180° phase angle shift, signifying their capacity for polarization switching. The butterfly-shaped loops characteristic of the filaments demonstrate the remarkable local piezoelectric effect.


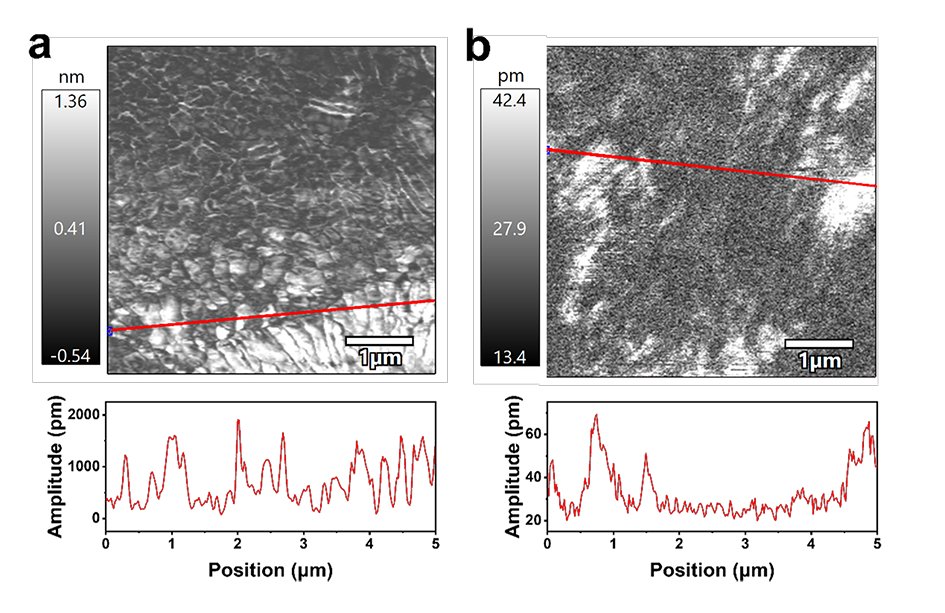


**Fig. S10** a) PFM amplitude (top panel) of the PZT-EGF composite filaments and amplitude curve (bottom panel) of the red line marked in top panel. b) PFM amplitude (top panel) of the PVDF matrix and amplitude curve (bottom panel) of the red line marked in top panel. Compared to PZT, PVDF exhibits significantly lower amplitude under the same driving voltage of 9 V (below 80 pm). This is equivalent to the noise level in PFM detection, hence the piezoelectricity of the PVDF matrix curing at 70 ℃ can be considered negligible when compared to PZT.


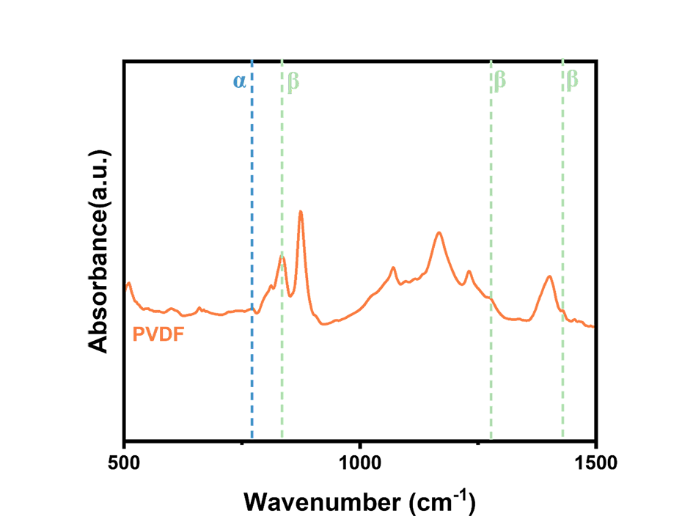


**Fig. S11** The FTIR spectra of PVDF matrix used for fixing filaments. The percentage content of β-phase in PVDF was calculated as the Lambert-Beer law (Note S1) according to the absorption vibrational bands at 762 cm^−1^ and 840 cm^−1^, yielded a percentage content of only 68.2% when curing at 70 ℃, which possess relatively lower piezoelectric properties cured at high temperature.


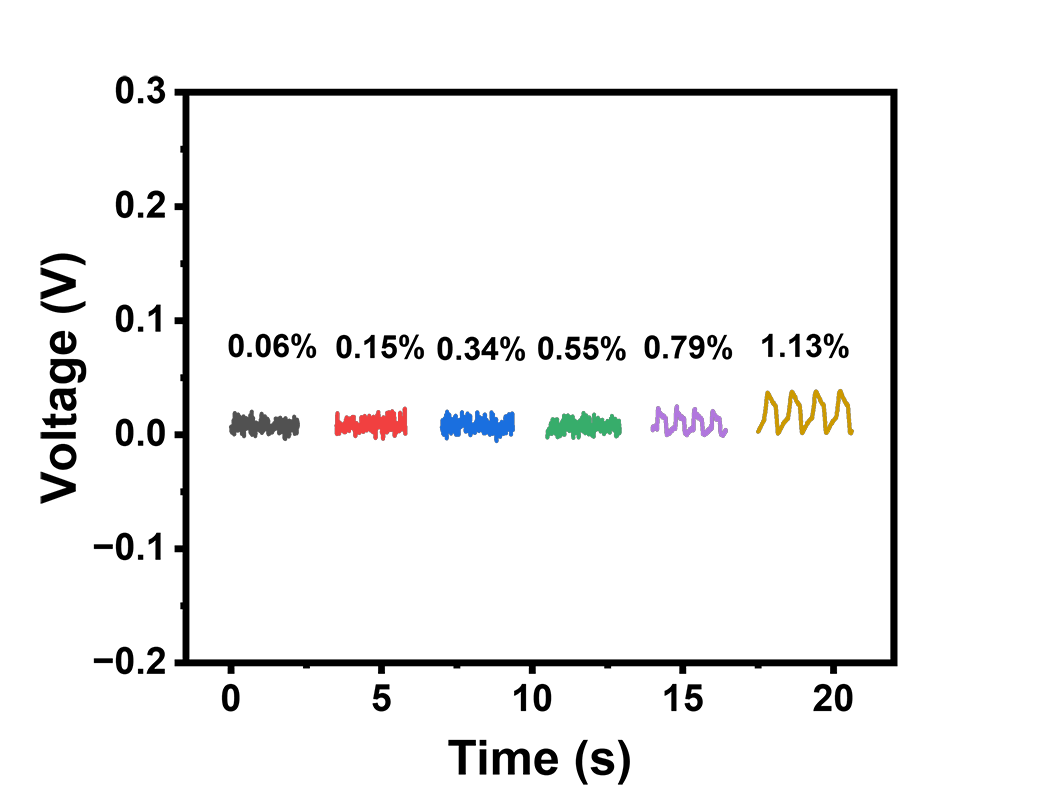


**Fig. S12** The output performance of the PVDF matrix.

The piezoelectric output of the device based on the pure PVDF film is less than 0.05 V across the strain range from 0.06% to 1.11%. In comparison to the vector sensor, the output of PVDF is negligible, indicating that the contribution of the PVDF matrix to the overall piezoelectric output of the vector sensor is minimal.


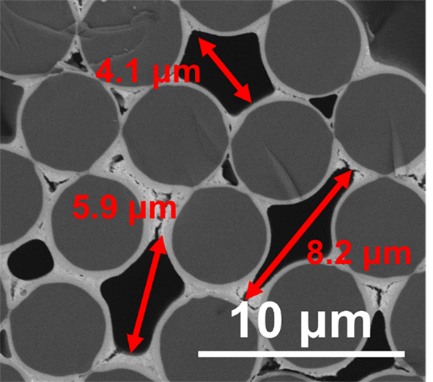


**Fig. S13** The size of the holes within the PZT-EGF composite fiber.


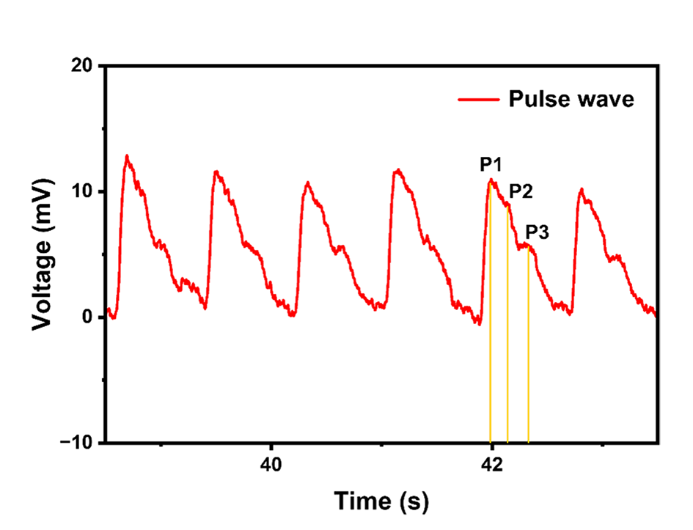


**Fig. S14** Real-time output voltage profiles of pulse for health monitoring. The pulse waveform clearly exhibits three distinct characteristic peaks (P1–P3), which correspond to the early systolic pressure peak (P1, representing the Percussion wave or P wave), the late systolic shoulder peak (P2, known as the Tidal wave or T wave), and the diastolic pulse peak (P3, also referred to as the Diastolic wave or D wave).


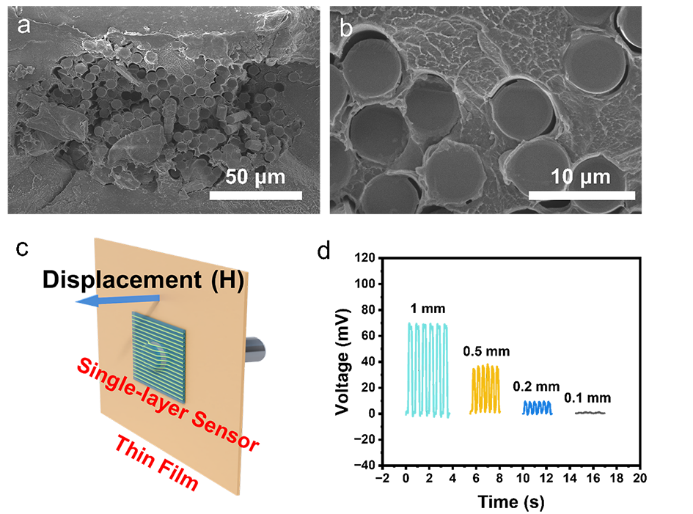


**Fig. S15** a) The SEM images of the anisotropic composite film without voids. b) Enlarged view of the inside of the filaments. c) Linear motor driving thin film micro-protrusions schematic diagram. d) The corresponding output voltage of the vector sensor without voids.


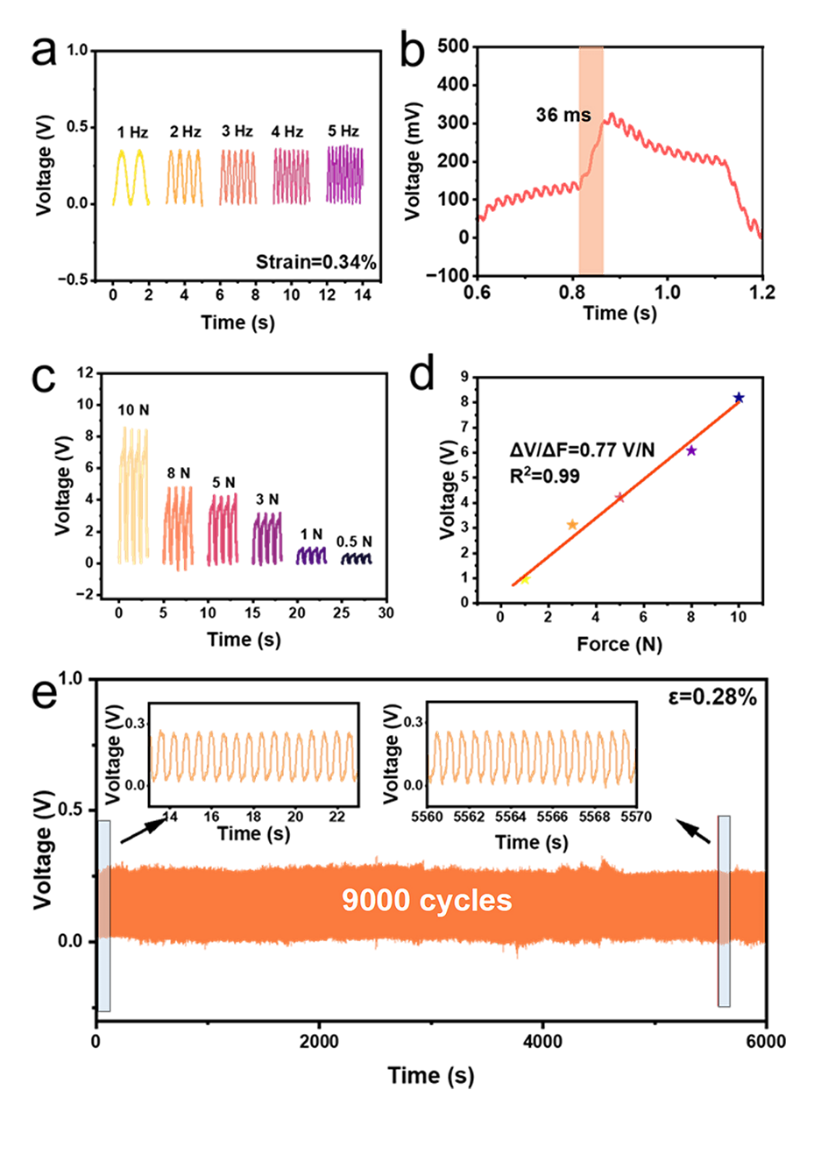


**Fig. S16** The comprehensive performance of the sensor. a) The Dependence of output voltage of vector sensor on the bending frequency along bending angle 0 degree. b) The response time of the output voltage under a 0.34% strain. c) Output voltage of the vector sensor with various tapping forces. d) Dependence of vector sensor output voltage on tapping forces. e) Change in output voltage over 9000 bending cycles under the strain of 0.28 %.

We measured the piezoelectric output of the device as it bent along 0 degrees with varying bending frequencies, as shown in Fig. S16a. It is noted that when the frequency changes from 1 Hz to 5 Hz, the piezoelectric output remains nearly constant, demonstrating the sensor's stability across different operating frequencies. The response speed of output voltage for external stimuli, defined as the elapsed time from the baseline to the peak, are only 36 ms (Fig.S16b). The output performances were standardized with various tapping forces applied to the sensor, in which a linear motor was used to apply forces accurately (Fig. S16c). Of note, there is a linear relationship between the applied forces and the output performance. Hence, the average voltages variation with forces are plotted in Fig. S16d. The sensitivity of the sensor is defined as the ratio of the change in output voltage (ΔV) and the change in force or stress (ΔF or Δσ). The sensitivity in this work is calculated by S = ΔV/ΔF. In the force range of 0.5–10 N, the voltage sensitivity is 0.77 V/N. Additionally, the sensor demonstrates excellent repeatability, as there is no noticeable degradation in performance after 9000 cycles (Fig. S16e).


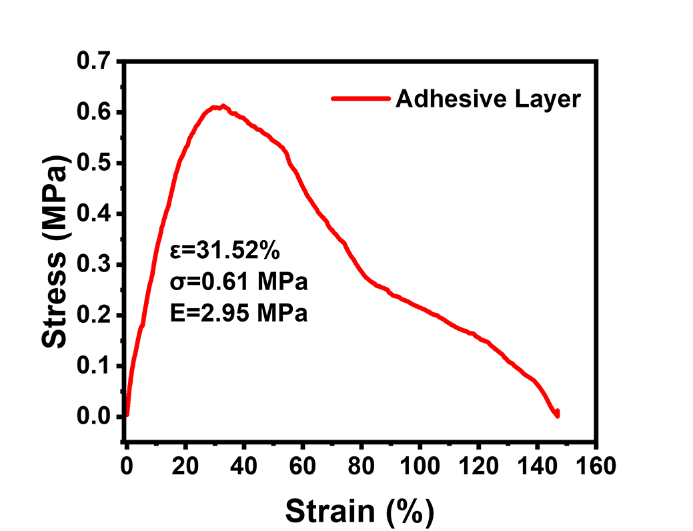


**Fig. S17** The mechanical performance of the adheisive layer of the double-layer sensors.

**
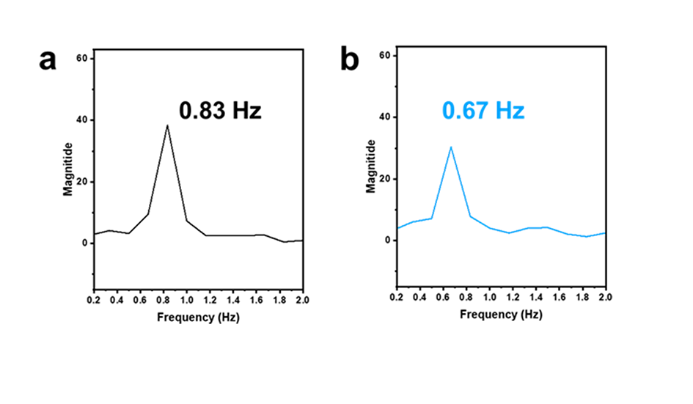
**

**Fig. S18** a) The frequency spectrum generated by FFT from the Figure 5c. b) The frequency spectrum generated by FFT from the Figure 5d.

**Table 1** The Mechanical Parameters of PVC Substrate

| **1** | **Tensile Yield Stress (MPa)** | **61.8** |
| --- | --- | --- |
| **2** | **Elongation at Break (%)** | **16** |
| **3** | **Tensile Elastic Modulus (MPa)** | **3250** |

**Table S2** Performance comparison with piezoelectric sensors reported in other works.

| Piezoelectric Materials | Sensitivity  (Tapping) | Response Time | Sensing Range (Strain/Bending radius） | Durability | Output Fluctuation | Ref |
| --- | --- | --- | --- | --- | --- | --- |
| **PZT/PVDF**  **(This work)** | **173 mV/kPa (770 mV/N）** | **36 ms** | **0.06%-1.11%（4.4cm-81.6cm）** | **9000** | **~1%** | **-** |
| P(VDF-TrFE) | - | - | 15 mm-35 mm | 1600000 | ~15.2% | [1] |
| PVDF | 7.7 mV /kPa | 10 ms | 10 mm-30 mm | 80000 | Little variation | [2] |
| ZnO NRs | - | - | 30 mm-50 mm | - | - | [3] |
| PZT | - | - | 0.2%-1% | 1000 | Little variation | [4] |
| PZT | - | - | 0.116%-0.214% | 15000 | Little variation | [5] |
| TPU/PVDF | 95.8 mV/N | 85 ms | - | 2000 | Little variation | [6] |
| PZT/PVDF | 1.28 mV/KPa | 21 ms | - |  |  | [7] |
| ZnO/PVDF | 3.12 mV/KPa | 53 ms | - | 5000 | Little variation | [8] |
| PDA/BTO/PVDF | 0.38 V/N | - | - | 7000 | Little variation | [9] |

**Reference:**

1. C. Yan, X. Li, X. Wang, G. Liu, Z. Yang, H. Tian, C. Wang, X. Chen, J. Shao. Extremely stable, multidirectional, all‐in‐one piezoelectric bending sensor with cycle up to million level. Adv. Funct. Mater., (2024). <https://doi.org/10.1002/adfm.202409093>

2. W. Lin, B. Wang, G. Peng, Y. Shan, H. Hu, Z. Yang. Skin-inspired piezoelectric tactile sensor array with crosstalk-free row+column electrodes for spatiotemporally distinguishing diverse stimuli. Adv Sci (Weinh), **8**(3), 2002817 (2021). <https://doi.org/10.1002/advs.202002817>

3. H.-J. Lee, S. Y. Chung, Y. S. Kim, T. I. Lee. Nonlinear piezoelectric dual sensor for the detection of angle and radius of a bending deformation. Nano Energy, **38**, 232-238 (2017). <https://doi.org/10.1016/j.nanoen.2017.05.053>

4. T. Sheng, Q. He, Y. Cao, Z. Dong, Y. Gai, W. Zhang, D. Zhang, H. Chen, Y. Jiang. Fish-wearable piezoelectric nanogenerator for dual-modal energy scavenging from fish-tailing. ACS Appl Mater Interfaces, **15**(33), 39570-39577 (2023). <https://doi.org/10.1021/acsami.3c08221>

5. Y. Liu, L. Ding, L. Dai, X. Gao, H. Wu, S. Wang, C. Zhuang, L. Cai, Z. Liu, L. Liu, J. Zhang, Y. Wang. All‐ceramic flexible piezoelectric energy harvester. Adv. Funct. Mater., **32**(52), 2209297 (2022). <https://doi.org/10.1002/adfm.202209297>

6. C. Wei, H. Zhou, B. Zheng, H. Zheng, Q. Shu, H. Du, A. Ma, H. Liu. Fully flexible and mechanically robust tactile sensors containing core–shell structured fibrous piezoelectric mat as sensitive layer. Chem. Eng. J., **476**, (2023). <https://doi.org/10.1016/j.cej.2023.146654>

7. G. Tian, W. Deng, Y. Gao, D. Xiong, C. Yan, X. He, T. Yang, L. Jin, X. Chu, H. Zhang, W. Yan, W. Yang. Rich lamellar crystal baklava-structured pzt/pvdf piezoelectric sensor toward individual table tennis training. Nano Energy, **59**, 574-581 (2019). <https://doi.org/10.1016/j.nanoen.2019.03.013>

8. T. Yang, H. Pan, G. Tian, B. Zhang, D. Xiong, Y. Gao, C. Yan, X. Chu, N. Chen, S. Zhong, L. Zhang, W. Deng, W. Yang. Hierarchically structured pvdf/zno core-shell nanofibers for self-powered physiological monitoring electronics. Nano Energy, **72**, (2020). <https://doi.org/10.1016/j.nanoen.2020.104706>

9. Y. Su, W. Li, L. Yuan, C. Chen, H. Pan, G. Xie, G. Conta, S. Ferrier, X. Zhao, G. Chen, H. Tai, Y. Jiang, J. Chen. Piezoelectric fiber composites with polydopamine interfacial layer for self-powered wearable biomonitoring. Nano Energy, **89**, (2021). <https://doi.org/10.1016/j.nanoen.2021.106321>

**Table S3** Performance comparison with sensors based on other principles

| **Device Structure** | | |
| --- | --- | --- |
| **Piezoelectric Device**  Simple structure; self-powered | **Piezoresistive Device**  Simple structure; need to be powered | **Triboelectric Device**  Self-powered;  The structure is complex, which requires both positive and negative electrodes, and the contact-separation mode devices occupy a large volume of space. |

| **Sensitivity** | **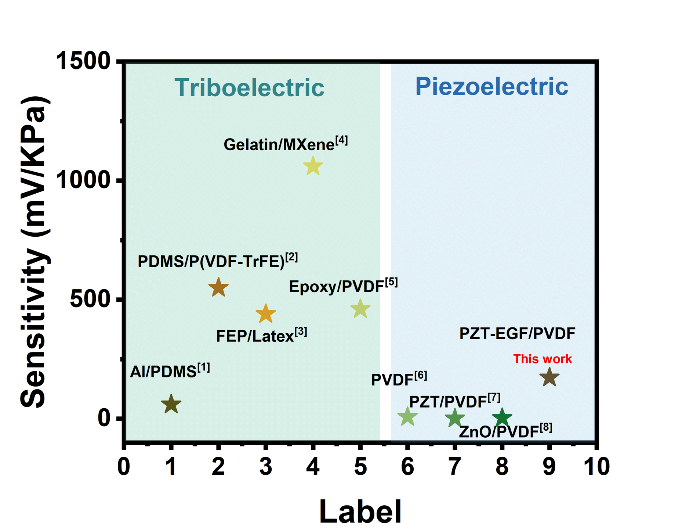** |
| --- | --- |
| **Reponse Time** | **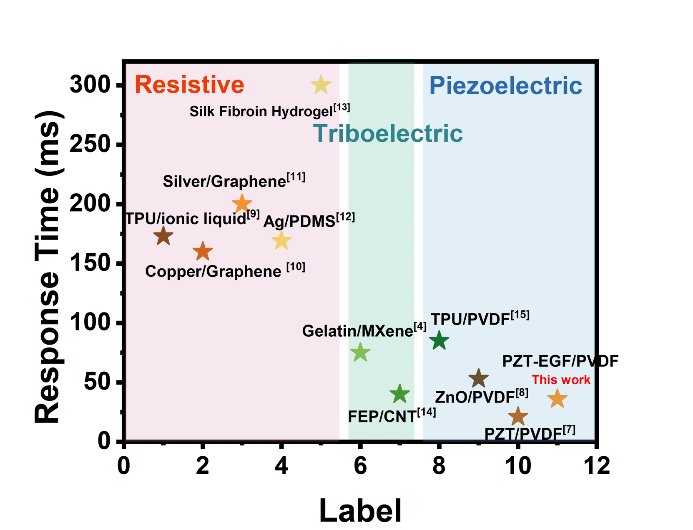** |
| **Durability** | **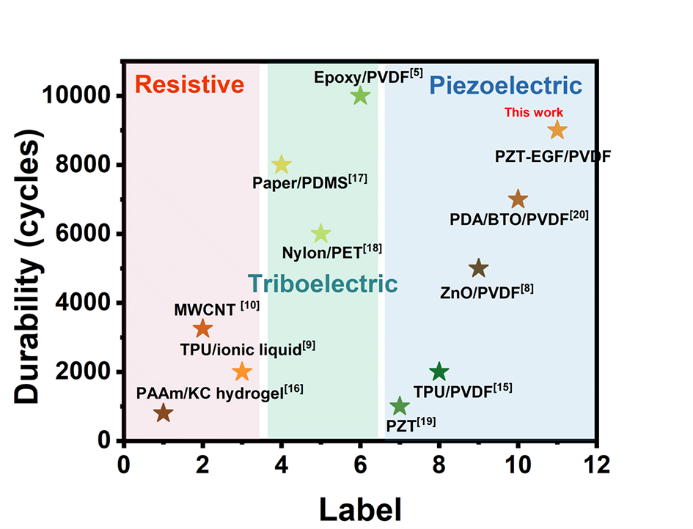** |

Overall, piezoelectric sensors have a simple structure and are self-powered, making them more advantageous for miniaturization compared to triboelectric sensors. As for sensitivity, the sensitivity of triboelectric sensors is greatly influenced by the device's structure (e.g., gap size, electrode materials), resulting in greater variability, and is generally slightly better than that of piezoelectric sensors^[21,22]^. However, due to the triboelectric effect, triboelectric sensors are highly susceptible to environmental influences, making them less stable compared to piezoelectric sensors. The sensitivity of piezoresistive sensors, measured by changes in resistance, cannot be directly compared to that of piezoelectric sensors^[23]^. Additionally, our sensor demonstrates a high sensitivity of 137 mV/kPa, which is considered high among piezoelectric devices. Regarding response time and durability, piezoelectric sensors clearly outperform piezoresistive sensors and are comparable to triboelectric sensors.

**Reference:**

1. X. Wang, H. Zhang, L. Dong, X. Han, W. Du, J. Zhai, C. Pan, Z. L. Wang. Self-powered high-resolution and pressure-sensitive triboelectric sensor matrix for real-time tactile mapping. Adv. Mater., **28**(15), 2896-2903 (2016). <https://doi.org/10.1002/adma.201503407>

2. M. Ha, S. Lim, S. Cho, Y. Lee, S. Na, C. Baig, H. Ko. Skin-inspired hierarchical polymer architectures with gradient stiffness for spacer-free, ultrathin, and highly sensitive triboelectric sensors. ACS Nano, **12**(4), 3964-3974 (2018). <https://doi.org/10.1021/acsnano.8b01557>

3. P. Bai, G. Zhu, Q. Jing, J. Yang, J. Chen, Y. Su, J. Ma, G. Zhang, Z. L. Wang. Membrane‐based self‐powered triboelectric sensors for pressure change detection and its uses in security surveillance and healthcare monitoring. Adv. Funct. Mater., **24**(37), 5807-5813 (2014). <https://doi.org/10.1002/adfm.201401267>

4. C. Zheng, D. Gao, B. Lyu, C. Zhang, H. Li, Y. Zhou, N. Li, J. Ma. A triboelectric sensor with highly sensitive and durable: Dual regulation strategy based on surface morphology and functional groups on negative/positive tribolayers. Chem. Eng. J., **477**, (2023). <https://doi.org/10.1016/j.cej.2023.147071>

5. Y. Fang, J. Xu, X. Xiao, Y. Zou, X. Zhao, Y. Zhou, J. Chen. A deep-learning-assisted on-mask sensor network for adaptive respiratory monitoring. Adv. Mater., **34**(24), e2200252 (2022). <https://doi.org/10.1002/adma.202200252>

6. W. Lin, B. Wang, G. Peng, Y. Shan, H. Hu, Z. Yang. Skin-inspired piezoelectric tactile sensor array with crosstalk-free row+column electrodes for spatiotemporally distinguishing diverse stimuli. Adv Sci (Weinh), **8**(3), 2002817 (2021). <https://doi.org/10.1002/advs.202002817>

7. G. Tian, W. Deng, Y. Gao, D. Xiong, C. Yan, X. He, T. Yang, L. Jin, X. Chu, H. Zhang, W. Yan, W. Yang. Rich lamellar crystal baklava-structured pzt/pvdf piezoelectric sensor toward individual table tennis training. Nano Energy, **59**, 574-581 (2019). <https://doi.org/10.1016/j.nanoen.2019.03.013>

8. T. Yang, H. Pan, G. Tian, B. Zhang, D. Xiong, Y. Gao, C. Yan, X. Chu, N. Chen, S. Zhong, L. Zhang, W. Deng, W. Yang. Hierarchically structured pvdf/zno core-shell nanofibers for self-powered physiological monitoring electronics. Nano Energy, **72**, (2020). <https://doi.org/10.1016/j.nanoen.2020.104706>

9. F. Wang, J. Chen, X. Cui, X. Liu, X. Chang, Y. Zhu. Wearable ionogel-based fibers for strain sensors with ultrawide linear response and temperature sensors insensitive to strain. ACS Appl Mater Interfaces, **14**(26), 30268-30278 (2022). <https://doi.org/10.1021/acsami.2c09001>

10. W. Zhu, C. Zhang, J. Lin, S. Pan, Q. Wang, N. Liao, P. Yu, M. Zhang. Flexible strain sensor based on copper/graphene composite films. ACS Applied Nano Materials, **7**(1), 358-369 (2023). <https://doi.org/10.1021/acsanm.3c04551>

11. C. Shen, C. Zhang, G. Cao, D. Liang, N. Liao. Waterproof strain sensor based on silver/graphene composite film for fine and large strain detection. Measurement, **239**, (2025). <https://doi.org/10.1016/j.measurement.2024.115482>

12. J. Wang, L. Liu, C. Yang, C. Zhang, B. Li, X. Meng, G. Ma, D. Wang, J. Zhang, S. Niu, J. Zhao, Z. Han, Z. Yao, L. Ren. Ultrasensitive, highly stable, and flexible strain sensor inspired by nature. ACS Appl Mater Interfaces, **14**(14), 16885-16893 (2022). <https://doi.org/10.1021/acsami.2c01127>

13. R. Wang, Q. Liu, J. Wei, C. Zhu, Y. Wang, A. Yu, W. Wang, J. Zou, J. Xie, Z. Fu. Biocomposite silk fibroin hydrogel with stretchability, conductivity and biocompatibility for wireless strain sensor. Journal of Materials Science & Technology, **210**, 195-203 (2025). <https://doi.org/10.1016/j.jmst.2024.05.021>

14. Y. Fang, Y. Zou, J. Xu, G. Chen, Y. Zhou, W. Deng, X. Zhao, M. Roustaei, T. K. Hsiai, J. Chen. Ambulatory cardiovascular monitoring via a machine-learning-assisted textile triboelectric sensor. Adv. Mater., **33**(41), e2104178 (2021). <https://doi.org/10.1002/adma.202104178>

15. C. Wei, H. Zhou, B. Zheng, H. Zheng, Q. Shu, H. Du, A. Ma, H. Liu. Fully flexible and mechanically robust tactile sensors containing core–shell structured fibrous piezoelectric mat as sensitive layer. Chem. Eng. J., **476**, (2023). <https://doi.org/10.1016/j.cej.2023.146654>

16. S. Lu, Z. Ma, X. Huang, Y. Wu, Z. Wang, J. Liu, M. Ding, L. Qin, G. Dong. Crack-based hydrogel strain sensors with high sensitivity and wide linear range. Chem. Eng. J., **496**, (2024). <https://doi.org/10.1016/j.cej.2024.153704>

17. Y. Zhong, J. Wang, L. Wu, K. Liu, S. Dai, J. Hua, G. Cheng, J. Ding. Dome-conformal electrode strategy for enhancing the sensitivity of batio(3)-doped flexible self-powered triboelectric pressure sensor. ACS Appl Mater Interfaces, **16**(1), 1727-1736 (2024). <https://doi.org/10.1021/acsami.3c14015>

18. J. Mao, P. Zhou, X. Wang, H. Yao, L. Liang, Y. Zhao, J. Zhang, D. Ban, H. Zheng. A health monitoring system based on flexible triboelectric sensors for intelligence medical internet of things and its applications in virtual reality. Nano Energy, **118**, (2023). <https://doi.org/10.1016/j.nanoen.2023.108984>

19. Y. Su, W. Li, L. Yuan, C. Chen, H. Pan, G. Xie, G. Conta, S. Ferrier, X. Zhao, G. Chen, H. Tai, Y. Jiang, J. Chen. Piezoelectric fiber composites with polydopamine interfacial layer for self-powered wearable biomonitoring. Nano Energy, **89**, (2021). <https://doi.org/10.1016/j.nanoen.2021.106321>

20. T. Sheng, Q. He, Y. Cao, Z. Dong, Y. Gai, W. Zhang, D. Zhang, H. Chen, Y. Jiang. Fish-wearable piezoelectric nanogenerator for dual-modal energy scavenging from fish-tailing. ACS Appl Mater Interfaces, **15**(33), 39570-39577 (2023). <https://doi.org/10.1021/acsami.3c08221>

21. S. K. Chittibabu, K. Chintagumpala, A. Chandrasekhar. Porous dielectric materials based wearable capacitance pressure sensors for vital signs monitoring: A review. Mater. Sci. Semicond. Process., **151**, (2022). <https://doi.org/10.1016/j.mssp.2022.106976>

22. X. Wang, J. Yu, Y. Cui, W. Li. Research progress of flexible wearable pressure sensors. Sensors and Actuators A: Physical, **330**, (2021). <https://doi.org/10.1016/j.sna.2021.112838>

23. L. Liu, M. Wu, W. Zhao, J. Tao, X. Zhou, J. Xiong. Progress of triboelectric nanogenerators with environmental adaptivity. Adv. Funct. Mater., **34**(7), (2023). <https://doi.org/10.1002/adfm.202308353>

**Video S1** Single-layer devices are utilized to detect isotropic micrometer-scale film deformations.

**Video S2** Double-layer devices are employed for detecting anisotropic micrometer-scale film deformations.
